# Predicting disease progression and poor outcomes in patients with moderately active rheumatoid arthritis: a systematic review

**DOI:** 10.1093/rap/rkz002

**Published:** 2019-02-15

**Authors:** Christopher J Edwards, Patrick Kiely, Subhashini Arthanari, Sandeep Kiri, Julie Mount, Jane Barry, Catherine R Mitchell, Polly Field, Philip G Conaghan

**Affiliations:** 1 NIHR Clinical Research Facility, University Hospital Southampton NHS Foundation Trust, Southampton; 2 Rheumatology Department, St George’s University Hospital NHS Foundation Trust, London, UK; 3 SAMETA (South Asia, Middle East, Turkey and Africa), Eli Lilly (Singapore) Pte Ltd, Singapore; 4 Health Outcomes and HTA Department; 5 Global Patient Outcomes and Real Word Evidence (GPORWE) International; 6 Medical Affairs, Eli Lilly and Company Ltd, Basingstoke; 7 Value Demonstration Practice, Oxford PharmaGenesis Ltd, Oxford; 8 Leeds Institute of Rheumatic and Musculoskeletal Medicine, University of Leeds and NIHR Leeds Biomedical Research Centre, Leeds, UK

**Keywords:** rheumatoid arthritis, moderate disease activity, disease progression, prognostic factors, systematic review

## Abstract

**Objectives:**

Access to biologic DMARDs for RA is often restricted to those with severe disease. This systematic review aimed to identify prognostic factors in patients with moderate disease activity who may be at risk of disease progression and poor clinical outcomes.

**Methods:**

MEDLINE, Embase and Cochrane databases were searched (final search 22 September 2017), and data from patients with moderate disease [28-joint DAS (DAS28) >3.2–≤5.1] were included. Studies were evaluated according to the measure(s) of progression/poor outcome used: radiographic, disease activity or other indicators.

**Results:**

The searches identified 274 publications, of which 30 were selected for data extraction. Fourteen studies were prioritized, because they specifically analysed patients with moderate RA. Nine studies reported radiographic progression outcomes for 3241 patients, three studies reported disease activity progression for 1516 patients, and two studies reported other relevant outcomes for 2094 patients. Prognostic factors with consistent evidence for progression/poor outcome prediction were as follows: DAS28 ≥ 4.2, the presence of anti-CCP antibodies, and power Doppler ultrasound score ≥1. Some predictors were specific to either disease activity or radiographic progression.

**Conclusion:**

Several criteria used in standard clinical practice were identified that have the potential to inform the selection of patients with moderate RA who are at greater risk of a poor outcome. A combination of two or more of these factors might enhance their predictive potential. Further work is required to derive clinical decision rules incorporating these factors.

**Key messages**

- Three prognostic factors were identified in moderately active RA patients at greater risk of disease progression.
- DAS28 ≥4.2, anti-CCP antibody presence and power Doppler ultrasound score ≥1 were identified as indicators of potential progression.
- Higher-risk RA patients with moderate disease activity may benefit from more intensive treatment strategies.

## Introduction

It is estimated that 47–53% of patients with early RA (<12 months since diagnosis) develop moderate to severe disease over 5 years [1], where moderate disease is defined as a 28-joint DAS (DAS28) of >3.2–≤5.1, and severe disease as a DAS28 > 5.1 [2]. Although moderately active RA is common, studies aiming to predict RA progression have largely included patient populations with early and severe RA [3].

Treatments for RA include conventional synthetic DMARDs (csDMARDs), biologic DMARDs (bDMARDs) and, more recently, targeted synthetic DMARDs (tsDMARDs). EULAR guidelines recommend that bDMARDS are used only in patients with poor prognostic factors, including moderate to severe disease, after the failure of one or more csDMARDs [4]. In England and Wales, the National Institute for Health and Care Excellence recommends that approved bDMARDs should be used for RA only when the disease activity is severe and has not responded to intensive therapy with a combination of csDMARDs [5]. Australian guidelines state that established severe or persistent disease must be present, or the patient should have had previous unsuccessful treatment with at least two csDMARDs [6]. These guidelines require patients to receive multiple csDMARDs before treatment with biologics. These requirements for high disease activity do not address the issue that many people with a moderate DAS may have disease progression, and therefore optimal treatment, for example, the use of bDMARDs, may be necessary for these individuals. The aim of this systematic literature review was to identify prognostic factors in patients with RA with moderately active disease who were at greater risk of progression and who had poor clinical outcomes.

## Methods

### Search strategy

MEDLINE, MEDLINE In-Process & Other Non-Indexed Citations, Embase and Cochrane Library databases were searched via Ovid on 27 October 2016 (final search 22 September 2017). The search strings and number of sources found are provided in Supplementary Table S1, available at *Rheumatology Advances in Practice* online. Inclusion criteria were limited to English-language publications, and no date restrictions were applied. Supplementary searches to identify relevant congress abstracts are outlined in Table 1. Abstracts and posters were screened online, and the bibliographies of eligible systematic reviews and meta-analyses were searched manually for relevant publications. Conference abstracts were included even if no subsequent publication was found, in order to capture as many data sources as possible.

**Table 1:** Congress proceedings searched as part of the systematic review

Number	Congress	Congress month	Proceedings available at time of search
1	International Society for Pharmacoeconomics and Outcomes Research (ISPOR), USA	May	2014–2017
2	ISPOR, Europe	October/November	2014–2017
3	European League Against Rheumatism (EULAR)	June	2014–2017
4	American College of Rheumatology (ACR)	November	2014–2017
5	British Society for Rheumatology (BSR) conference	April	2014–2017

### Citation screening, full text review and data extraction

One analyst screened the title and abstract of the retrieved references to determine whether they met the predefined eligibility criteria (Table 2); any uncertainties were resolved by a second analyst. Publications that met the inclusion criteria were re-assessed against the review criteria. Data were extracted into predefined data summary tables and checked by a second independent analyst.

**Table 2:** Eligibility criteria for inclusion in the systematic review

Eligibility criteria	
Population	Patients with active RA Moderate or severe stage (DAS28 >3.2)^a^ Adults (aged ≥18 years)
Interventions	Any or no intervention (patients not treated with biologics were prioritized)
Outcomes	Predictive or prognostic factors that may influence patient outcomes. Examples are listed below. Demographic markers ○Age, BMI, disease duration, sex, smoking status Clinical markers ○DAS ○Duration of morning joint stiffness ○Extra-articular manifestations ○HAQ score ○Joint erosion ○Patient VAS in DAS28 ○Symmetrical polyarthritis Imaging markers ○Radiographic score at baseline ○MRI or ultrasound features at baseline Inflammatory markers ○CRP ○ESR Genetic markers ○*PTPN22* gene ○HLA-DRB1 shared epitope Presence of autoantibodies ○Anti-CCP antibody ○Anti-peptidyl-arginine deiminase-4 antibody ○IgA RF Bone markers ○Cartilage oligomeric matrix protein ○Collagen cross-linked C-telopeptide ○Human cartilage glycoprotein-39 ○MMP-3 ○RANK ligand:osteoprotegerin ratio The influence of these factors on the following patient outcomes was assessed HAQ (patient assessment of functional ability), arthritis impact measurement scales, McMaster–Toronto arthritis questionnaire scores Radiographic progression Change in DAS or DAS28 ACR 20/ACR 50/ACR 70 response EULAR response Remission Patient assessment of pain (using VAS or Likert scale) EQ-5D score Patient/physician assessment of disease activity (using VAS or Likert scale) Morning stiffness, number of flares
Study design	No restriction
Publication type^b^	Primary Pooled data Systematic review and meta-analysis (included in order to search reference lists)
Date restriction	No date restriction
Language restriction	English only (non-English-language publications with an English abstract were considered for inclusion)
Country	No restriction (European and US publications were prioritized)

a Studies that recruited only patients with moderate RA were prioritized at the final selection stage; therefore, studies with a mixed RA population were deprioritized.

b General narrative reviews, editorials, economic analysis and cost studies were excluded.

Abbreviations: ACR 20/50/70: 20%/50%/70% improvement in ACR criteria; DAS28: 28-joint DAS; EQ-5D: European quality of life – five dimensions; VAS: visual analog scale.

### Definitions of progression

We used several progression outcomes. These are summarized across three categories (radiographic progression, disease activity progression and other) in Supplementary Table S2, available at *Rheumatology Advances in Practice* online. A well-established way of measuring structural disease progression is achieved by radiographic assessment. Most studies used the van der Heijde–Sharp (vdHS) score (a minimum increase of one, three or five units, although not consistent across studies). Some used other radiographic scores, such as the Larsen or Ratingen score, or MRI scores. These are described in detail in Supplementary Table S3, available at *Rheumatology Advances in Practice* online.

**Table 3:** Summary of outcomes, studies and findings in moderate RA populations

Factor	Reference(s)	Results
**Radiographic progression**		
PDUS	Moller *et al*., 2016 [27]	Patients with PDUS scores above thresholds of 6/66 (20%), 4/66 (30%) and 2/66 (50%) were significantly more likely to experience future radiographic progression over 5 years of follow-up (*P* < 0.05)
	De Miguel *et al*., 2015 [14]	PDUS score ≥1 at baseline (OR, 5.067; 95% CI, 1.162, 21.576; *P* = 0.017) and remaining at 6 months (OR, 7.474; 95% CI, 2.644, 21.123; *P* < 0.0005) was associated with radiographic progression
GSUS	Moller *et al*., 2016 [27]	Patients with GSUS score above thresholds of 18/66 (20%), 16/66 (30%) and 11/66 (50%) were significantly more likely to experience radiographic progression over the 5-year follow-up period (*P* < 0.05)
	Sundlisater *et al*., 2016 [31]	GSUS score predicted radiographic progression (OR, 1.03 per point, *P*=0.019)
Specific damage measured by imaging	McQueen *et al*., 2014 [24]	Specific damage at baseline (radial osteitis, synovitis at the radioulnar, radiocarpal and intercarpal–carpometacarpal joints) was predictive for the Auckland magnetic resonance imaging cartilage score after 3 years of follow-up (*P* < 0.005)
vdHS score	Sundlisater *et al*., 2016 [31]	Total vdHS predicted radiographic progression (OR, 1.08, *P* = 0.017)
	Alemao *et al*., 2014 [8]	Baseline vdHS score was associated with rapid radiographic progression at 2 years of follow-up (OR, 1.01; 95% CI, 1.00, 1.01)
	Fautrel *et al.*, 2015 [3]	Patients with moderate RA receiving MTX treatment may exhibit radiographic progression, particularly those with both high CRP and RF+ at baseline, after 2 and 3 years of MTX treatment
Antibody status	Alemao *et al*., 2014 [8]	RF or anti-CCP antibody presence was predictive of rapid radiographic progression (measured by change in vdHS score) at 2 years (OR, 3.35; 95% CI, 1.41, 7.99)
	Alemao *et al.*, 2016 [7]	Presence of anti-CCP but not RF was predictive for erosive disease [OR, 2.72 (95% CI, 1.77, 4.18) and 1.36 (95% CI 0.88, 2.08), respectively] and for low disease activity (SDAI <3.3) [0.37 (95% CI, 0.21, 0.66) and 1.45 (95% CI 0.82, 2.56), respectively]
	Kroot *et al*., 2000 [21]	Presence of anti-CCP antibodies at baseline predicts development of joint erosions (OR, 2.72; 95% CI, 1.77, 4.18)
	Barra *et al*., 2013 [10]	Presence of anti-CCP antibodies at baseline was significantly associated with disease progression after 6 years of follow-up
Genetic biomarkers	Li *et al*., 2016 [22]	The frequency and severity of radiographic progression increased as MBDA scores became higher within the high range (17.4 for change in vdHS score >5 with MBDA score ≥60). In multivariate analyses, MBDA score had the most significant association with radiographic progression (*P* = 0.002 for change in vdHS score >3; *P* = 0.005 for change in vdHS score >5). When conventional risk factors, such as SJC, CRP and DAS28-CRP, were low, MBDA score significantly differentiated risk for progression
**Disease progression**		
DAS28	Kiely *et al*., 2011 [20]	DAS28 <3.2 at end of year 2 was achieved in 37% of patients with low-moderate disease at year 1, and in 16% of patients with high-moderate disease (OR, 3.12; 95% CI, 1.5, 6.6). DAS28 <3.2 at the end of year 3 was achieved in 48% of patients with low-moderate disease and in 19% with high-moderate disease (OR, 4.06; 95% CI, 1.7, 9.7). Patients were significantly less likely to achieve a low HAQ score (<1.25) at year 2 than those with a year 1 DAS28 <3.2. Note: this study was in a mixed population, but stratified by disease activity; therefore, the moderate RA subgroup can be described as a standalone
	Mogosan *et al.*, 2016 [26]	There are significant predictive relationships between DAS28, HAQ and EQ-5D scores: linear regression modelling showed that DAS28 is strongly predicted by EQ-5D (*P* < 0.0001), with >50% of the variability of DAS28 being determined by the variability of EQ-5D. HAQ score has a predictive value for DAS28 (*P* < 0.0001), with ∼30% of the variability of DAS28 being determined by the variability of HAQ
**Other progression indicators**		
Initiation of biologics	Bykerk *et al*., 2010 [11]	DAS28 at baseline was the only independent factor predicting the initiation of biologics within 1 year (OR, 1.48; *P* < 0.000001)
Requirement for joint surgery	Nikiphorou *et al.*, 2015 [28]	Patients with high-moderate DAS28 (4.1–5.1) at baseline had a significantly higher risk of requiring intermediate surgery within 5 years (HR, 1.80; 95% CI, 1.05, 3.11, *P*=0.034) than those with low-moderate (>3.2–4.19) or low DAS28 (>2.6–3.2). Patients with DAS28 in the low-moderate or high-moderate range at baseline had a significantly higher risk of requiring major surgery (large joint replacements) within 5 years than those with low DAS28 (HR, 2.07; 95% CI, 1.28, 3.33; *P* < 0.005 and HR, 2.16; 95% CI, 1.32, 3.52; *P* < 0.005, respectively). The rate of surgery at 5 years was significantly higher in patients with high-moderate DAS (4.2–5.1) after 1 year than in those with low DAS (>2.6–3.2; *P* < 0.05)

Abbreviations: DAS28: 28-joint DAS; DAS28-CRP: 28-joint DAS using CRP; EQ-5D: European quality of life – five dimensions; GSUS: grey-scale ultrasound; HR: hazard ratio; MBDA: multi-biomarker disease activity; OR: odds ratio; PDUS: power Doppler ultrasound; SDAI: simplified disease activity index; vdHS: van der Heijde–Sharp.

Disease activity progression was typically defined using standard DAS28 thresholds (remission, <2.6; low, ≤3.2; moderate, >3.2–≤5.1; severe, >5.1). Some studies used a Health Outcomes Questionnaire (HAQ) to define progression in disability. Full details for each study are presented in Supplementary Table S4, available at *Rheumatology Advances in Practice* online. The ‘other outcomes’ category included surrogate factors, such as initiation of biologics or the requirement for major joint surgery, alongside more standard health-related quality of life measures. Full details of the studies in this category are provided in Supplementary Table S5, available at *Rheumatology Advances in Practice* online.

**Table 4:** Identified prognostic factors and reported thresholds for patients with moderate RA

Factor	Threshold for progression	Sources
DAS28	>4.2 at baseline	Kiely *et al*., 2011 [20]
		Nikiphorou *et al.*, 2015 [28]
Presence of anti-CCP antibodies	(Presence at baseline)	Alemao *et al*., 2014 [8]
		Alemao *et al.*, 2016 [7]
		Kroot *et al*., 2000 [21]
		Barra *et al*., 2013 [10]
PDUS	PDUS score ≥1 at baseline	De Miguel *et al*., 2015 [14]

Abbreviations: DAS28: 28-joint DAS; PDUS: power Doppler ultrasound.

## Results

### Search results

**Fig. 1:**
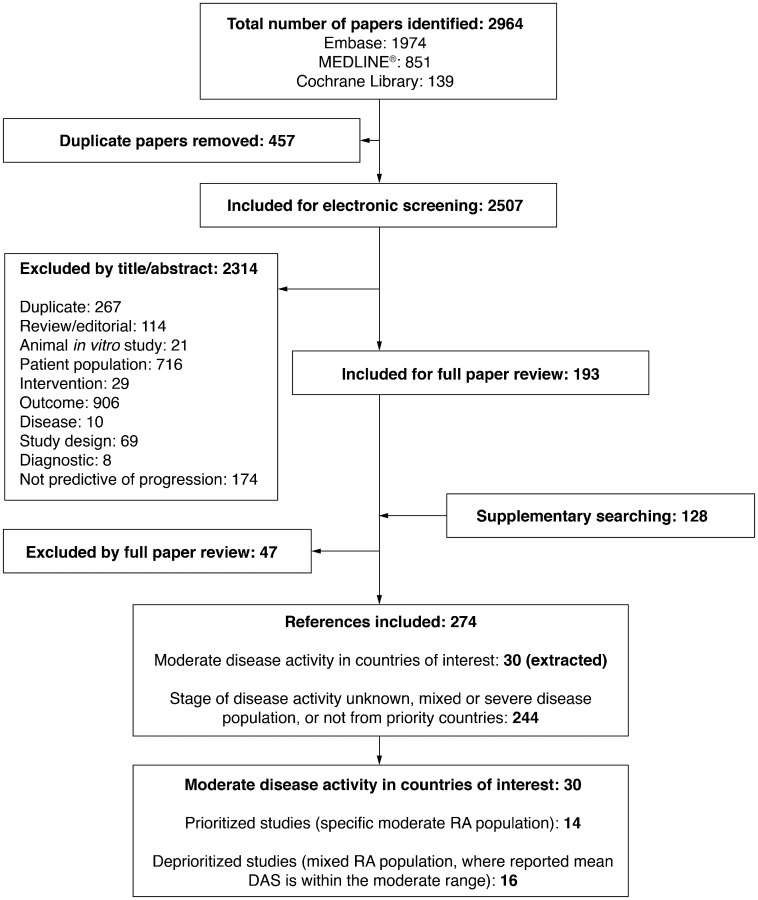
PRISMA flow diagram PRISMA: preferred reporting items for systematic reviews and meta-analyses.

The database searches identified 2964 articles, 457 of which were duplicates, leaving 2507 articles for electronic screening; 2314 were excluded after applying inclusion and exclusion criteria. Thus, 193 underwent full review, and 47 were excluded. A further 128 relevant congress abstracts were identified, giving 274 total references that met the broad inclusion criteria. Details are shown in the PRISMA flow diagram in Fig. 1.

Only studies that reported data from study populations with moderate disease activity were included, allowing focus on the identification of markers for greater risk of progression in patients with moderate RA. Data from 30 sources [7–35] were extracted. We further prioritized studies where the study population was explicitly limited to patients with moderate RA, leaving 14 prioritized sources [3, 7, 8, 10, 11, 14, 20–22, 24, 26–28, 31], and deprioritizing 16 studies [9, 12, 13, 15–19, 23, 25, 29, 30, 32–35] that had populations with mixed RA disease activity. These deprioritized studies that reported a mean DAS within the moderate range are summarized in Supplementary Table S6, available at *Rheumatology Advances in Practice* online.

### Summary of prioritized moderate RA population studies

Several potential prognostic markers for disease progression were examined across the 14 prioritized publications. We summarized the evidence for the association between these markers and each of the three categories of progression outcome: radiographic progression, disease activity and functional progression or other progression measures (initiation of biologics and need for joint surgery) (Supplementary Table S2, available at *Rheumatology Advances in Practice* online), and summarized the prognostic markers investigated in each study and the overall study outcome (Table 3).

Three prognostic factors were identified in moderately active RA patients at greater risk of disease progression: DAS28 ≥ 4.2, the presence of anti-CCP antibodies and power Doppler ultrasound score (PDUS) ≥ 1. These thresholds are summarized in Table 4.

### Radiographic progression

The factors below were assessed for their ability to predict radiographic progression, defined as changes in vdHS score or Ratingen score, or increase in cartilage damage. The methodology of the included radiographic progression studies is shown in Supplementary Table S3, available at *Rheumatology Advances in Practice* online, and the results for each study are provided in Supplementary Table S7, available at *Rheumatology Advances in Practice* online.

#### Power Doppler ultrasound and grey scale ultrasound

Two studies, one Swiss (*n *=* *377) and one Spanish (*n *=* *129), examined the prognostic value of PDUS in moderate disease. In the Swiss study, patients had radiographic damage to their hands appraised by a blinded assessor. Forty-nine per cent of the study population had received bDMARDs. Radiographic damage progression was defined as an increase in the Ratingen score (range 0–190; smallest detectable change 3.3 points). Twenty-two joints were assessed, with each joint being scored from 0 to 3. Score thresholds of 6/66, 4/66 and 2/66 were used to define the worst 20, 30 and 50% of PDUS scores, respectively. Patients with scores above these thresholds were significantly more likely to experience radiographic progression over the 5-year follow-up period (univariate analyses) [27].

In the Spanish study, radiographs of hands and feet were scored according to vdHS scores. Radiographic progression was assessed in 12 joints (scored 0–3), and progression was deﬁned as an increase of more than one point. A PDUS score >1 at baseline was associated with radiographic progression [odds ratio (OR), 5.067; 95% CI, 1.162, 21.576; *P *=* *0.017], as was maintenance of that score over 1 at 6 months (OR, 7.474; 95% CI, 2.644, 21.123; *P *<* *0.001) [14].

A similar result was found for grey scale ultrasound (GSUS) damage scores, with two publications [one from Switzerland (*n *=* *377) [27] and one from the Netherlands (*n *=* *222) [31] in patients with moderate disease activity] reporting that baseline damage scores could significantly predict disease progression. The Swiss study assessed 22 joints (scored from 0 to 3) and used GSUS score thresholds of 18/66, 16/66 and 11/66 to define the worst 20, 30 and 50% of GSUS scores, respectively. A significantly higher proportion of patients (*P *<* *0.05) with a GSUS score greater than each of these thresholds had radiographic progression over the 5 years of follow-up (univariate analyses) [27].

In the Dutch study, patients were followed up for 24 months and assessed using vdHS scores. Progression was defined as an increase of more than one point per year. In the multivariate model, RF positivity (OR, 2.27, *P *=* *0.022), total vdHS (OR, 1.08, *P *=* *0.017) and GSUS score (OR, 1.03 per point, *P *=* *0.019) were independent baseline predictors of radiographic progression at 24 months [31].

#### Magnetic resonance imaging

MRI-detected inflammatory pathologies at baseline (baseline radial osteitis, synovitis at the radioulnar, radiocarpal and intercarpal–carpometacarpal joints) were shown to be predictive for the Auckland cartilage score after 3 years of follow-up (*P *<* *0.005) in an MRI study of patients with moderate RA (*n *=* *28) [24].

#### vdHS score

Three studies examined the prognostic value of baseline vdHS score as a marker of radiographic progression [3, 8, 31]. In an American prospective observational study of 644 patients with moderate RA (95% DMARD naïve), baseline vdHS score was one of several markers that were associated with radiographic progression, as measured by changes in vdHS score at 2 years (OR, 1.01; 95% CI, 1.00, 1.01) [8]. Other markers were seropositivity for RF or anti-CCP antibody, duration of RA of <2 years, below normal body weight and DAS28. However, in a French study of 96 patients with moderate RA, the baseline erosion score was not predictive of significant radiographic progression (vdHS score >3.0 overall) over 3 years of follow-up [3]. In a Dutch study (*n *=* *222; methodology described above) [31], RF positivity (OR, 2.27; *P *=* *0.022), total vdHS (OR, 1.08; *P *=* *0.017) and GSUS score (OR, 1.03 per point; *P *=* *0.019) were independent baseline predictors of radiographic progression at 24 months in a multivariate model [31].

#### Antibody status

Antibodies were investigated as potential prognostic markers for radiographic progression in several studies. An American study in 644 patients with moderate RA reported that seropositivity for either RF or anti-CCP antibody at baseline predicted rapid radiographic progression, as measured by changes in vdHS score at 2 years (OR, 3.35; 95% CI, 1.41, 7.99) [8]. Another American study (*n *=* *1309) found that anti-CCP antibodies at baseline in patients with moderate disease predicted the development of joint erosions (OR, 2.72; 95% CI, 1.77, 4.18) [7]. The presence of anti-CCP antibodies and erosions (*vs* absence) was associated with a greater extent of erosions/joint deformity and lower odds of remission [7]. Two further studies report that patients with anti-CCP antibodies had developed significantly more severe radiological damage than those without [10, 21].

#### Biomarkers

A retrospective observational study reported that the multi-biomarker disease activity (MBDA) score enhanced the ability of conventional risk factors [i.e. serological status, swollen joint count (SJC), CRP and DAS28-CRP (modified DAS28 using CRP)] to predict radiographic progression in patients with moderate RA receiving non-biologic DMARDs [22].

### Disease activity and functional progression

Factors assessed for their ability to predict progression in disease activity (measured by DAS28 or HAQ score) are detailed below. The methodology of these studies is shown in Supplementary Table S4, available at *Rheumatology Advances in Practice* online and the results in Supplementary Table S8, available at *Rheumatology Advances in Practice* online.

#### DAS28 at baseline

An assessment of the use of DAS28 at baseline was performed in 418 patients with newly diagnosed RA stratified by DAS28 (low, <3.2; low-moderate, 3.2–4.1; high-moderate, 4.2–5.1; high >5.1) 1 year after presentation. The proportions of patients with a year 1 high-moderate DAS28 who achieved DAS28 < 3.2 at year 2 (16%) and year 3 (19%) were similar to the proportions of patients with a year 1 high DAS28 (>5.1) who achieved this outcome (13 and 15%, respectively). Patients with a year 1 moderate DAS28 (3.2–5.1) were significantly less likely to achieve a low HAQ score (<1.25) at year 2 than those with a year 1 DAS28 < 3.2 [20].

#### Anti-CCP antibody status

The presence of anti-CCP antibodies as a predictor of disease activity progression was assessed in one multinational study (Canada, USA and UK) in patients with moderate RA (*n *=* *342) but reported that the presence of anti-CCP antibodies did not predict disease progression after 2 years, as measured by HAQ score, DAS28 or SJC [10].

### Other progression indicators

Other RA progression indicators are summarized below. The methodology of the studies included in this category is shown in Supplementary Table S5, available at *Rheumatology Advances in Practice* online, and the outcomes are detailed in Supplementary Table S9, available at *Rheumatology Advances in Practice* online.

#### Initiation of biologics

In a Canadian prospective observational study in 1146 patients with moderate RA, DAS28 at baseline was the only independent factor predicting the initiation of biologics, a proxy of disease progression, within 1 year (OR, 1.48, *P *<* *0.001) [11].

#### Requirement for joint surgery

An examination of the ability of baseline DAS28 to predict whether joint surgery would be required within 5 years in a population with moderate RA (mean DAS28, 4.8) was performed [28]. Patients with high-moderate (4.2–5.1, *n *=* *426) baseline DAS28 had a significantly higher risk of requiring intermediate surgery (synovectomies and arthroplasties of wrist/hand or hind/forefoot) within 5 years than those with low-moderate (>3.2–4.19, *n *=* *522) or low (>2.6–3.2) DAS28 [hazard ratio (HR), 1.80; 95% CI, 1.05, 3.11; *P *=* *0.034]. Patients with low-moderate or high-moderate DAS28 had a significantly higher risk of requiring major surgery (defined as large joint replacements) within 5 years than those with low DAS28 (HR, 2.07; 95% CI, 1.28, 3.33; *P *<* *0.005 and HR, 2.16; 95% CI, 1.32, 3.52; *P *<* *0.005, respectively). The rate of such surgery at 5 years was significantly higher in patients with high-moderate DAS (4.2–5.1) after 1 year than in those with low DAS (>3.2–4.19; *P *<* *0.05) [28].

## Discussion

This review identified evidence supporting the use of higher levels within the moderate DAS28 range (DAS28 >3.2–≤5.1), imaging (PDUS, GSUS or MRI), anti-CCP antibody status and MBDA tests to identify patients who are at greater risk of progression and poorer clinical outcomes than other patients in this category. It should be noted that a moderate DAS28 level does not equate to a benign outcome, and these patients may potentially benefit from more intensive treatment strategies, because there is a low likelihood of moderate patients achieving a low DAS28 with csDMARDs [20]. Some of the identified progression measures are already used routinely in the clinic, and using the results for predicting prognosis should impose a minimal implementation cost. Although MRI and MBDA tests are not currently used in standard practice, there is evidence of their value as prognostic factors.

In terms of radiographic progression, higher baseline DAS28 within the moderate range was commonly reported to predict poor outcomes [10, 20, 26]. In particular, higher moderate baseline DAS28 (range, 4.2–5.1) was able to predict the requirement for intermediate (synovectomies and arthroplasties of wrist/hand or hind/forefoot) or major joint surgery within 5 years [28], and patients with early moderate RA who did not achieve remission after a year of DMARD treatment were very unlikely to achieve remission within 3 years [20].

There is strong evidence that imaging results are prognostic of radiographic progression, shown in six studies [8, 14, 24, 27, 31]. The imaging results included PDUS [14, 27] and GSUS scores [27, 31] and specific measures of damage, such as baseline radial osteitis and synovitis [24], in addition to vdHS [8]. The anti-CCP antibody was significantly associated with severe radiological damage after 6 years of follow-up, and RF or anti-CCP antibodies at baseline were predictive of radiographic progression [8]. The MBDA score enhanced the ability of conventional risk factors (i.e. serological status, SJC, CRP and DAS28-CRP) to predict radiographic progression [22].

This systematic review also assessed prognostic factors for disease activity progression. Patients with low or low-moderate baseline DAS28 were more likely to achieve a DAS28 <3.2 over 2 years of follow-up, although these findings are from a single study [20]. The anti-CCP antibody was also significantly associated with disease progression in one study [10].

Several studies suggested that the value of these prognostic factors could be enhanced by using them in combination. Fautrel *et al.* [3] concluded that high CRP levels combined with RF positivity at baseline were a strong indicator for radiographic progression at 2 and 3 years of follow-up. A mixed population study (deprioritized in this review) reported that DAS28 could be combined with up to three risk factors (anti-CCP antibodies, ESR and Ratingen score) to predict joint damage progression more accurately than DAS28 alone [15]. The authors of both studies suggested that optimizing treatment for patients with poor prognostic outcomes would include earlier intervention with bDMARDs. Further studies are required to establish the sensitivity of these factors, either alone or in combination, and to define which patients would most benefit from earlier bDMARD intervention.

This systematic review has some limitations. Publications were identified by searching for keywords, such as ‘moderate’ and ‘predictor’, in the title and abstract. Therefore, relevant publications that did not contain these keywords might have been missed. Our search identified publications that reported a median or mean DAS28 in the moderate range, but in fact consisted of mixed RA populations. Therefore, these might include some outlying patients with mild or severe disease activity. We deprioritized these studies, halving the potential number of publications available to analyse for this review. More evidence might have been available for populations with moderate disease, but if the publication did not report a baseline DAS28 value or did not label the disease severity as ‘moderate’, the study would not have been included for extraction. Additionally, radiographic disease progression is demonstrated in most of the studies using the Sharp van Der Heijde score, with several different thresholds reported. Some are below the minimally clinically relevant value (typically five) and, as a result, a degree of caution is required in interpreting these studies. Finally, we were not able to conduct any quantitative synthesis of the data, because the progression measures, study designs and markers explored were too heterogeneous.

In summary, several factors, potentially in combination, can identify patients with moderate RA who are at risk of disease progression and a poor clinical outcome. These patients should be considered as candidates for escalation of therapy to bDMARDs or tsDMARDs, using a treat-to-target approach. The heterogeneity of studies found in this systematic review showed that there is not currently a robust algorithm in place to identify patients with moderate RA who are at greater risk of disease progression; therefore, further work is required to develop clinical decision rules to identify these patients. A real-world evidence study would be the preferred approach here, assessing a combination of prognostic factors that could be used to develop a predictive tool for clinical use.

## Supplementary Material

Supplementary Data
