# Supplementary material for: Predicting disease progression and poor outcomes in patients with moderately active rheumatoid arthritis: a systematic review
Source: Rheumatol Adv Pract. 2019 Feb 15;3(1):rkz002. doi: 10.1093/rap/rkz002 (PMC6649936; doi:10.1093/rap/rkz002)
Supplement: Supplementary Data [file rkz002_supp.docx]

# Appendix 1

SUPPLEMENTARY TABLE S1 Electronic search strategy

### **Embase, conducted on 27 October 2016**

| [**#**](http://ovidsp.tx.ovid.com/sp-3.24.1b/ovidweb.cgi?&S=ODCIFPIKAGDDIAPDNCHKEADCOKHEAA00&Sort+Sets=descending) | **Searches** | **Results** |
| --- | --- | --- |
| 1 | rheumatoid arthritis.mp. or rheumatoid arthritis/ | 187 434 |
| 2 | (moderate or severe or mixed or (DAS28 adj2 "3")).tw. | 1 681 103 |
| 3 | (stage* adj (II or "2" or III or "3")).tw. | 96 599 |
| 4 | 2 or 3 | 1 766 311 |
| 5 | 1 and 4 | 19,526 |
| 6 | ((prognos* or predict* or risk or prospective) adj3 (factor* or indicator* or marker* or measurement* or outcome* or variable* or criteri*or scor*or characteristic* or finding* or value* or observation*)).tw. or risk factor/ or predictive value/ or observer variation/ | 1 625 087 |
| 7 | 5 and 6 | 2331 |
| 8 | limit 7 to english language | 2203 |
| 9 | limit 8 to ("conference review" or editorial or "review") | 229 |
| 10 | 8 not 9 | 1974 |

### **MEDLINE, conducted on 27 October 2016**

| [**#**](http://ovidsp.tx.ovid.com/sp-3.24.1b/ovidweb.cgi?&S=ODCIFPIKAGDDIAPDNCHKEADCOKHEAA00&Sort+Sets=descending) | **Searches** | **Results** |
| --- | --- | --- |
| 1 | rheumatoid arthritis.mp. or Arthritis, Rheumatoid/ | 118 977 |
| 2 | (moderate or severe or mixed or (DAS28 adj2 "3")).tw. | 1 277 701 |
| 3 | (stage* adj (II or "2" or III or "3")).tw. | 62 453 |
| 4 | 2 or 3 | 1 333 388 |
| 5 | 1 and 4 | 9906 |
| 6 | ((prognos* or predict* or risk or prospective) adj3 (factor* or indicator* or marker* or measurement* or outcome* or variable* or criteri*or scor*or characteristic* or finding* or value* or observation*)).tw. or Risk Factors/ or Predictive Value of Tests/ or Observer Variation/ | 1 392 644 |
| 7 | 5 and 6 | 1128 |
| 8 | limit 7 to english language | 1025 |
| 9 | limit 8 to (editorial or "review") | 174 |
| 10 | 8 not 9 | 851 |

### **Cochrane, conducted on 27 October 2016**

|  | **Searches** | **Results** |
| --- | --- | --- |
| 1 | rheumatoid arthritis.mp. or Arthritis, Rheumatoid/ | 8619 |
| 2 | (moderate or severe or mixed or (DAS28 adj2 "3")).tw. | 111 262 |
| 3 | (stage* adj (II or "2" or III or "3")).tw. | 8501 |
| 4 | 2 or 3 | 118 223 |
| 5 | 1 and 4 | 1550 |
| 6 | ((prognos* or predict* or risk or prospective) adj3 (factor* or indicator* or marker* or measurement* or outcome* or variable* or criteri*or scor*or characteristic* or finding* or value* or observation*)).tw. or Risk Factors/ or Predictive Value of Tests/ or Observer Variation/ | 79 293 |
| 7 | 5 and 6 | 337 |
| 8 | limit 7 to english language [Limit not valid in CDSR,ACP Journal Club,DARE,CLCMR; records were retained] | 334 |
| 9 | limit 8 to (editorial or "review") [Limit not valid in CDSR,ACP Journal Club,DARE,CLCMR,CLHTA,CLEED; records were retained] | 195 |
| 10 | 8 not 9 | 139 |

### **Outcome measures overview**

SUPPLEMENTARY TABLE S2 Baseline biomarker and disease progression measures

| **Baseline biomarker** | **Radiographic progression** | | | | | **Disease activity** | | **Other** | | |
| --- | --- | --- | --- | --- | --- | --- | --- | --- | --- | --- |
|  | Change in vdHS score | Change in Larsen score | Change in Ratingen score | Change in RAMRIS score | Increase in cartilage damage | Change in DAS28 | Change in HAQ score | Intermediate or major joint surgery | Extent of erosions/joint deformity | Use of biologics |
| **Clinical** | | | | | | | |  | | |
| Age | 🗶[3]  🗶[31] | ****^a^[33]  🗶^b^[33] |  |  |  | ****^a^[33]  🗶^b^[33] | ****[25]  ****^d^[33]  🗶^b^[33]  ****[21] |  |  | 🗶[11] |
| Sex | 🗶[3]  🗶[31] |  |  |  |  |  | 🗶[25]  ****[21] |  |  |  |
| Educational status |  |  |  |  |  |  | 🗶[25] |  |  |  |
| Socioeconomic status |  |  |  |  |  |  |  |  |  | 🗶[11] |
| Smoking status | 🗶[31] | 🗶[33] |  |  |  | 🗶[33] | 🗶[25]  🗶[33] |  |  | 🗶[11] |
| BMI | 🗶[31] |  |  |  |  |  | 🗶[25] |  |  |  |
| Underweight | ****[8] |  |  |  |  |  |  |  |  |  |
| Duration of RA | ****[8]  🗶[3]  🗶[31] | 🗶[33] |  |  |  | 🗶[26]  ****^b^[33]  🗶^a^[33] | 🗶[33] |  |  | 🗶[11] |
| Year of RA onset |  |  |  |  |  |  | 🗶[25] |  |  |  |
| Any comorbidities |  |  |  |  |  |  | 🗶[25] |  |  |  |
| Heart disease |  |  |  |  |  |  | ****[25] |  |  |  |
| Hypertension |  |  |  |  |  |  | ****[25] |  |  |  |
| Severity of disease |  |  |  |  |  |  | 🗶[25] |  |  |  |
| SDAI score | 🗶[14] |  |  |  |  |  |  |  |  |  |
| CDAI score | 🗶[14] |  |  |  |  |  |  |  |  |  |
| M-DAS28 | 🗶[12] |  |  |  |  |  |  |  |  |  |
| M-SDAI score | 🗶[12] |  |  |  |  |  |  |  |  |  |
| M-CDAI score | 🗶[12] |  |  |  |  |  |  |  |  |  |
| DAS28 | ****[8]  🗶[14] | ****^a^[33]  🗶^b^[33] |  |  |  | ****[20]  ****[33]  🗶[9] | ****[20]  ****[33]  ****[21] | ****[28] |  | ****[11] |
| EQ-5D score |  |  |  |  |  | ****[26] |  |  |  |  |
| HAQ score |  | 🗶[33] |  |  |  | ****[33]  ****[26] | ****[33] |  |  |  |
| Anxiety and depression |  |  |  |  |  | ****^d^[35] |  |  |  |  |
| Grip strength |  |  |  |  |  |  |  |  |  |  |
| Joint count | 🗶[31] |  |  |  |  |  |  |  |  | ****[11] |
| Morning stiffness |  |  |  |  |  |  |  |  |  |  |
| ESR | ****[3]  🗶[31] |  |  |  |  |  |  |  |  | 🗶[11] |
| CRP | ****[3]  🗶[32] | ****^a^[33]  🗶^b^[33] |  | 🗶[32] |  | 🗶[33] | 🗶[33] |  |  |  |
| Pain |  |  |  |  |  |  |  |  |  |  |
| FDI |  |  |  |  |  |  |  |  |  |  |
| **Bone or imaging markers** | | | | | | | | | | |
| PDUS score | ****[14] |  | ****[27] |  |  |  |  |  |  |  |
| GSUS score | ****[31] |  | ****[27] |  |  |  |  |  |  |  |
| Osteitis |  |  |  |  | ****[24] |  |  |  |  |  |
| Synovitis |  |  |  |  | ****[24] |  |  |  |  |  |
| vdHS | ****[8]  🗶[3]  ****[31] |  |  |  |  |  |  |  |  |  |
| Radiological changes/erosions at baseline | 🗶[3]  ****[21] | ****[33] |  |  |  | 🗶[9] | 🗶[33] |  |  | 🗶[11] |
| Larsen score |  | ****[33] |  |  |  | 🗶[33] | 🗶[33] |  |  |  |
| **Genetic markers** | | | | | | | | | | |
| 17 risk alleles | ****^e^[34] |  |  |  |  |  |  |  |  |  |
| Reported heredity |  | 🗶[33] |  |  |  | 🗶[33] | 🗶[33] |  |  |  |
| Presence of ‘shared epitope’ |  | 🗶[33] |  |  |  | 🗶[33] | ****^a^[33]  🗶^b^[33] |  |  |  |
| **Serum inflammatory or bone destruction markers** | | | | | | | | | | |
| sCTx-I | ****[32] |  |  | ****[32] |  |  |  |  |  |  |
| uCTx-II | ****[32] |  |  | 🗶[32] |  |  |  |  |  |  |
| sOPG | 🗶[32] |  |  | 🗶[32] |  |  |  |  |  |  |
| sCOMP | 🗶[32] |  |  | 🗶[32] |  |  |  |  |  |  |
| sYKL-40 | 🗶[32] |  |  | 🗶[32] |  |  |  |  |  |  |
| sMMP-3 | 🗶[32] |  |  | 🗶[32] |  |  |  |  |  |  |
| IL-15 |  |  |  |  |  | ****[17] |  |  |  |  |
| NLR |  |  |  |  |  | 🗶[29] | 🗶[29] |  |  |  |
| **Autoantibody markers** | | | | | | | | | | |
| Anti-CCP antibody | ****[8]  ****[3]  🗶[31]  ****[21] |  |  |  |  | ****[17]  🗶[10]  ****[16]  ****[30] | 🗶[10]  🗶[16] |  | ****[17]  🗶[10]  ****[7]  ****[16] | 🗶[11] |
| RF | ****[8]  ****[3]  ****[31]  ****[21] | ****[33] |  |  |  | ****^b^[33]  🗶^a^[33]  🗶[9]  🗶[10] | 🗶[33]  🗶[10]  ****[21] |  | 🗶[10]  🗶[7] | 🗶[11] |
| Anti-sa |  | ****[19] |  |  |  |  |  |  |  |  |
| **Matrices or composite markers** | | | | | | | | | | |
| MBDA | ****[18]  ****[22]  ****[23] |  |  |  |  | 🗶[9] |  |  |  |  |
| ASPIRE CRP | 🗶[13] |  |  |  |  |  |  |  |  |  |
| ASPIRE ESR | 🗶[13] |  |  |  |  |  |  |  |  |  |
| BeSt | 🗶[13] |  |  |  |  |  |  |  |  |  |
| SWEFOT1 | 🗶[13] |  |  |  |  |  |  |  |  |  |
| SWEFOT2 | 🗶[13] |  |  |  |  |  |  |  |  |  |
| ESPOIR | 🗶[13] |  |  |  |  |  |  |  |  |  |
| Anti-CCP antibody, high ESR and erosions (number of risk factors) |  |  | ****[15] |  |  |  |  |  |  |  |

^a^In women only.

^b^In men only.

^c^Measured by the Arthritis Impact Measurement Scales.

^d^Measured by EQ-5D.

^e^17 gene alleles comprising *rs4810485*, *rs7667746*, *rs7665842*, *rs4371699*, *rs6821171*, *rs1896368*, *rs1896367*, *rs1528873*, *rs2104286*, *s8192916*, *rs1119132*, *rs7607479*, *rs26232*, *rs11908352*, *rs451066*, *rs1465788*, *rs1485305*.

****, baseline value predictive of future disease progression; : baseline value not predictive of future disease progression.

ASPIRE: Active-Controlled Study of Patients Receiving Infliximab for the Treatment of Rheumatoid Arthritis of Early Onset; BeST: Behandel Strategieen; BMI: body mass index; CCP: cyclic citrullinated peptide; (M-) CDAI: (modified) Clinical Disease Activity Index; CRP: C-reactive protein; (M-) DAS28: (modified) 28-joint Disease Activity Score; EQ-5D: European Quality of Life-5 dimensions; ESPOIR: Etude et Suivi des POlyarthrites Indifférenciées Récentes; ESR: erythrocyte sedimentation rate; FDI: Functional Disability Index; GSUS: grey-scale ultrasound; HAQ: Health Assessment Questionnaire; IL: interleukin; MBDA: multi-biomarker disease activity; NLR: neutrophil:lymphocyte ratio; PDUS: power Doppler ultrasound; PRO: patient-reported outcome; RA: rheumatoid arthritis; RAMRIS: Rheumatoid Arthritis Magnetic Resonance Imaging Scoring System; RF: rheumatoid factor; (M-) SDAI: (modified) Simplified Disease Activity Index; sCOMP: serum cartilage oligomeric matrix protein; sCTx-I: serum C-terminal cross-linking telopeptide of type I collagen; sMMP: serum matrix metalloproteinase; sOPG: serum osteoprotegerin; SWEFOT: Swedish Farmacotherapy; sYKL-40: human cartilage glycoprotein 39; uCTx-II: urine C-terminal cross-linking telopeptide of type II collagen; vdHS: van der Heijde–Sharp.

### **Radiographic progression: study information**

SUPPLEMENTARY TABLE S3 Radiographic progression: study information

| **Reference** | **Patient inclusion criteria** | ***N*** | **Mixed population/ moderate only^a^** | **DMARD history** | **Population characteristics** | **Markers assessed** | **Outcomes assessed/definition of progression** | **Duration of follow-up** |
| --- | --- | --- | --- | --- | --- | --- | --- | --- |
| Alemao *et al*., 2014[8]  USA  Prospective observational cohort study  Abstract | RA patients   - Patients from the Brigham and Women’s Hospital Rheumatoid Arthritis Sequential Study (BRASS) registry | 644 | Moderate only  Baseline mean DAS28: 3.9 | (5.0) | - Mean age (SD), years:  57 (14) - Female: 82% - Symptom duration, years: 15 - DAS28-CRP, mean (SD): 3.9 (1.6) - Total swollen tender joints, mean (SD): 16.1 (14.1) - vdHS, mean (SD): 48.6 (61.1) - RF+ or anti-CCP+: 71% | Variables including   - Duration of RA - Under to normal weight - DAS28-CRP - RF+ or anti-CCP+ - vdHS | Annualized change in vdHS score calculated; patients with ≥5 unit increase was considered rapid progressors | NR |
| Alemao *et al.,* 2016[7]  USA  Registry study  Abstract | Established RA patients | 1309 | Moderate only,  Baseline DAS28-CRP, mean (SD): 3.7 (1.6) | % naïve/ treated, NR  Anti-CCP+ patients, with erosions (*n*=498), 60% treated with bDMARDs  All other patients, 38% treated with bDMARDs | - Female: 82% - Mean age (SD), years: 56.5 (14.1) - Mean TJC (SD): 14.3 (14.0) - Mean anti-CCP+ (SD), units/mL: 128.3 (151.9) - Mean RF levels (SD), units/mlL 127.7 (301.6) | - Anti-CCP - RF | Presence of erosions and disease activity (not defined) | NR |
| Bakker *et al*., 2012[9]  USA  Prospective cohort study  Full publication | RA patients   - From CAMERA cohort - 1987 ACR criteria for RA - Age >16 years - Treated with MTX ± ciclosporin | 74 | Mixed population  Baseline DAS28, mean (SD): 5.6 (1.0) | MTX, 100% | - Serum samples from subgroup of patients in the CAMERA cohort - Female: 52 (70%) - Mean (SD) age, years: 53 (15) - RF+: 50 (68%) - TJC28, mean (SD): 9 (6) - SJC28, mean (SD): 10 (5) - ESR, mean (SD), mm/h: 39 (29) | - MBDA test | Clinical disease activity as measured by DAS28-CRP  Radiographic progression, >0 vdHS units over 2 years | 2 years |
| Couderc *et al*.,  2015[12]  France  Prospective cohort study  Abstract | RA patients   - Patients from the multicentre ESPOIR cohort - Patients fulfilling the ACR/EULAR 2010 classification criteria for RA - Disease duration <6 months | 590 | Mixed population  Baseline DAS28-ESR, mean: 5.2 | NR | - Mean age, years: 48.8 - Female: 78% - Disease duration, weeks: 16 - SJC, mean: 8 - CRP, mg/L: 22 - ESR at first hour, mm: 29 - DAS28-CRP, mean:5.0 - SDAI: 30.4 - CDAI: 28.3 | Variables including:   - M-DAS28 - M-SDAI - M-CDAI | Radiographic progression estimated by vdHS scoring system | 1 year |
| De Cock *et al*., 2014[13]  Belgium  Prospective observational cohort study  Full publication | RA patients   - Consecutive DMARD-naïve, early RA patients - Patients enrolled in parallel RCTs were excluded | 74 | Mixed population  Baseline DAS28-CRP, mean (SD): 4.91 (1.22) | Naïve, 100% | - Mean age (SD), years: 52 (16) - Female: 65% - Smoker: 28% - Symptom duration (SD), months: 8 (7) - Disease duration (SD), months: 1 (1) - RF+: 71% - ESR (SD), mm/h: 36.66 (24.06) - CRP (SD), mg/L: 28.80 (33.78) - Total TJC (SD): 14.35 (10.59) - Total SJC (SD): 12.45 (8.19) - HAQ score (SD): 1.1 (0.76) | Six matrices including:   - ASPIRE CRP (SJC [0–28], RF [U/L], CRP) - ASPIRE ESR (SJC [0–28], RF [U/L], ESR) - BeST (RF, anti CCP, CRP, erosions) - SWEFOT1 (RF, anti CCP, ESR, sex) - SWEFOT2 (CRP, erosions, smoker) - ESPOIR (SJC [0–28], anti-CCP, CRP, erosions) | RRP, defined as a vdHS score progression ≥5 | 2 years |
| De Miguel *et al*., 2015[14]  Spain  Prospective observational study  Abstract | RA patients   - Moderate disease activity (DAS28-CRP 3.2–5.1) - Started daily anti-TNF therapy - Patients with doubtful radiographic progression (progression = 1) excluded | 129 | Moderate only  ‘RA patients with moderate disease activity (DAS28-CRP 3.2–5.1) | NR | - Median age (IQR), years: 56 (44–66) - Female: 82.9% - Median time from diagnosis (IQR), years: 5.0 (3.0–11.5) - RF+: 63.6% | Reduced 12-joint PDUS examination score  DAS28 (ESR/CRP), SDAI, CDAI, and ACR/EULAR remission criteria | Radiographic progression defined as >1 point increase according to vdHS | 6–12 months |
| de Punder *et al*., 2015[15]  Netherlands  Retrospective observational cohort study  Full publication | RA patients   - Nijmegen early RA cohort - Met 1987 ACR criteria for RA - Disease duration <1 year at study entry - ≥18 years of age - Patients treated with bDMARD in the first 3 years excluded | 260 stratified by number of risk factors (Rfs):  0 Rfs, 31  1 Rf, 79  2 Rfs, 86  3 Rfs, 64 | Mixed population  Baseline DAS (SD):  0 Rfs, 3.5 (1.1)  1 Rf, 3.6 (1.3)  2 Rfs, 4.0 (1.1)  3 Rfs, 4.6 (0.9) | Naïve, 100% | - Mean age (SD), years: 0 Rfs, 56 (14) 1 Rf, 54 (14) 2 Rfs, 55 (14) 3 Rfs, 54 (14) - Female: 0 Rfs, 61% 1 Rf, 63% 2 Rfs, 55% 3 Rfs, 61% - RF+, n (%): 0 Rfs, 6 (19) 1 Rf, 56 (71) 2 Rfs, 76 (88) 3 Rfs, 91 (58) | Presence or absence of anti-CCP, high ESR and erosions was translated into four risk profiles: 0, 1, 2 and 3 | Structural damage progression assessed via Ratingen score; disease activity assessed by DAS over 3 years | 3 years |
| del Val del Amo *et al*., 2006[16]  Spain  Prospective cross-sectional study  Full Publication | RA patients   - Met 1997 ACR criteria for RA - Selection was made consecutively, taking into account DAS28 in order to include patients with all levels of disease activity | 89 | Mixed population  Baseline mean (SD), 3.96 (1.46)  Moderate, DAS28 3.2–≤5.1: 40 (45%) | Naïve, 0%  DMARDs unspecified | - Mean age (SD), years: 61 (13) - Female: 71.9% - Disease duration (SD), years: 7.6 (8.8) - ESR, mm/h, mean (SD): 28 (21) - CRP, mg/L, mean (SD): 16.1 (19.7) - M-HAQ score, mean (SD): 0.53 (0.46) - Erosions: 69.7% | Autoantibody biomarkers   - Anti-CCP | - ESR - CRP - RF - DAS28 - M-HAQ score - Simplified vdHS score (SENS)   Progression not defined | Cross-sectional study |
| Fautrel *et al*., 2015[3]  France  RCT  Full publication | RA patients   - Subanalysis of TEMPO trial (NCT00393471) - Data were used from the MTX arm of TEMPO for patients with sustained moderate RA (mean DAS28 ≥3.2–≤5.1) during the last 6 months of the first year | 96 | Moderate only  ‘moderate RA defined as ≥3.2 mean DAS28 ≤5.1’ | NR | - Mean age (SD), years: 54.0 (12.9) - Female: 82% - Disease duration (SD), years: 6.8 (5.5) - Total TJC (SD): 32.8 (13.3) - Total SJC (SD): 22.0 (10.2) - ESR (SD), mm/h: 41.8 (26.9) - CRP (SD), mg/L: 26.9 (29.0) - HAQ score (SD): 1.7 (0.6) - M-vdHS score (SD): 34.4 (51.7) - MTX dose, g/week: 7.40 (0.62) | One matrix with variables including ESR, RF positivity and CRP | Significant radiographic progression defined as vdHS score >3.0 overall | 3 years |
| Hambardzumyan *et al*., 2015[18]  Sweden  Randomized open-label efficacy study  Full publication | RA patients   - Enrolled in SWEFOT trial (NCT00764725) - DMARD-naïve patients with early RA (duration <12 months) - All patients received initial 3-months MTX monotherapy; MTX continued in all patients as part of a multiple therapy | 235 | Mixed population  Baseline DAS28, mean (SD): 5.7 (1.02)  Baseline DAS28-CRP, mean (SD): 5.4 (0.99) | Naïve, 100% | - Female: 72% - Symptom duration (SD), months:  6.1 (5.1) - SJC (0–28), mean (SD): 10.8 (5.31) - TJC (0–28), mean (SD): 9.3 (5.86) - CRP, mg/L, mean (SD): 35.4 (38.37) - ESR, mm/h, mean (SD): 41.3 (26.9) - MBDA score, mean (SD):  59.6 (14.71) - vdHS, mean (median): 4.7 (2) | - DAS28-ESR - DAS28-CRP - CRP - MBDA scores (biomarkers) | Radiographic progression defined as progression of vdHS score >5 points/year | 1 year |
| Hayem *et al*., 1999[19]  France  Prospective cohort study  Full publication | RA patients   - Met 1987 ACR criteria for RA | 154 | Mixed population (DAS NR) | NR | - Stratified into long-standing (*n* = 107) and recent onset (*n* = 47) - Anti-Sa antibodies: 39.8% | Autoantibody biomarkers   - Anti-CCP - RF - AKA - APF - Anti-RA33 - HLA class II DRB alleles - Anti-sa | Radiographic progression was defined as radiographic severity score using the Larsen scoring system at three sites: wrists, hands and feet, and one other site | 6 years |
| Kroot *et al*., 2000[21]  Netherlands  Prospective longitudinal cohort study  Full publication | Recent-onset RA patients   - Met 1987 ACR criteria for RA - Symptom duration <1 year at study entry | 273  Anti-CCP+, 179  Anti-CCP−, 94 | Moderate only  Baseline mean (SD) DAS:  Anti-CCP+, 4.1 (1.14)  Anti-CCP−, 4.2 (1.14) | Naïve, 100% | - Stratified by presence of anti-CCP - Mean age (SD), years: anti-CCP+, 51.1 (15.1); anti-CCP−, 52.4 (14.8) - Female: anti-CCP+, 62%; anti-CCP−, 73% - Mean HAQ (SD): anti-CCP+, 0.71 (0.43); anti-CCP−, 0.72 (0.42) | Autoantibody biomarkers   - Anti-CCP - IgM-RF positivity - HLA-DR4 positivity - Radiological score at entry - DAS - Age (years) at entry - Sex | Physical disability via HAQ score and radiographic progression via the modified vdHS score over 3 and 6 years. | 3 and 6 years |
| Li *et al*., 2016[22]  Netherlands  Retrospective observational study  Full publication | RA patients   - Using 1987 ACR criteria for RA - Disease activity generally moderate, median DAS28-CRP, 3.3; CRP, 0.8 mg/dL; mean MBDA score, 43 (moderate = 30–44)   All taking non-biologic DMARDs but not bDMARDs at enrolment | 163 | Moderate only  Baseline DAS28-CRP, median (IQR): 3.3 (2.3–4.3) | Conventional DMARDs, 100% | - Mean age (SD), years: 55 (14) - Female: 67% - Median disease duration, years: 4.6 - RF+: 66% - SJC28,0-28, median (IQR): 1 (0–4) - MBDA score (SD), mean: 43 (15) - CRP, median, mg/dL: 0.8 (0.3–1.7) - vdHS, median (IQR): 23 (11–47) | Six variables over 1 year   - MBDA score - SJC28 score - DAS28-CRP - CRP - Total vdHS score - Serological status | Radiographic progression defined as yearly progression of vdHS score >3.0 U or >5.0 U | 1 year |
| Markusse *et al*., 2014[23]  Netherlands  RCT  Full publication | RA patients   - Fulfilled 1987 criteria of ACR - Data for this *post hoc* analysis from the BeSt study (NTR262 and NTR265) | 125 | Mixed population  Baseline DAS28, mean (SD) 5.82 (1.03) | Naïve, 100% | - Female: 75.2% - Mean (SD) age, years: 53 (14) - TJC28, median (IQR): 11 (7–15.5) - Erosions, median (IQR): 1.0 (0.5–3.0) | - Twelve serum biomarkers measured to determine MBDA scores - DAS | Radiographic progression assessed using the vdHS; defined as increase in vdSH in the year after biomarker measurement | 2 years |
| McQueen *et al*., 2014[24]  New Zealand  Prospective cohort study  Full publication | RA patients   - Enrolled with early disease (Early) and established RA (Late) | 28  Early, 15  Late, 13 | Moderate only  DAS28 median (range): early, 3.1 (1.5–4.8); late, 3.6 (1.9–4.8) | % treated/ naïve, NR  bDMARDs: early, 1 (7); late, 4 (31)  Conventional DMARDs: totals NR^b^ | - Stratified into early and late RA - Median age (range), years: early, 57 (36–87); late RA 69 (43–84) - Female: early, 11 (73%); late RA, 8 (62%) - Median disease duration (range), years: early, 54 (48–72); late, 240 (83–456) - RF+: early, 60%; late, 69% - Anti-CCP: early, 93%; late, 92% - Median TJC (range): early, 8 (1–36); late, 17 (0–40) - Median SJC (range): early, 1 (0–6); late 3 (0–8) - Median HAQ (range): early, 0.75 (0–1.75); late, 0.88 (0–2.5) | MRI parameters such as AMRICS, OMERACT, MRI JSN, X-ray JSN | Cartilage damage via synovitis, osteitis, bone erosion and cartilage loss; progression based on AMRICS | 3 years |
| Moller *et al*., 2016[27]  Switzerland  Prospective observational cohort study  Abstract | RA patients  Patients in the SCQM registry with at least one X-ray before and after first ultrasound assessment with at least 20 of the recommended 22 joints evaluated in the SONAR score | 377 | Moderate only  DAS28-ESR median at baseline: 3.4 | % naïve, NR  Synthetic DMARDs, 66%  bDMARDS, 49% | - Median disease duration (mean ± SD), years: 6.4 (9.5 ± 9.8) - Median Ratingen score: 9.0 | Standardized GSUS and PDUS | Radiographic damage progression defined as increase in Ratingen score (range, 0–190) beyond the smallest detectable change of 3.3 score points | 5 years |
| Sundlisater *et al*., 2016[31]  Netherlands  Prospective observational study  Abstract | RA patients   - Disease duration  <2 years - 2010 ACR/EULAR criteria - DMARD naïve | 222 | Moderate only  ‘Mean DAS based on 44 joints was 3.5 [SD, 1.2]’ | 222 (100) | - Disease duration (SD), months: 7.2 (5.4) - DAS, mean (SD): 3.5 (1.2) - RF+, 72% - Anti-CCP+, 82% | Demographic variables   - Sex - Age - Smoking status - RF - Tender joints - 44 SJC - ESR - GSUS - PDUS - vdHSs - BMI - Disease duration <3 months - Anti-CCP - Patient global score | Radiographs scored by two readers using the vdHS, with cut-off ≥1 unit increase/year classified as progression | 24 months |
| Syversen *et al*., 2009[32]  Norway  Prospective longitudinal cohort study  Full publication | Early RA patients   - Disease duration  <1 year | 84 | Mixed population  Baseline DAS28, median (IQR), 4.2 (3.0–5.1) | Naïve, 22.6%  Conventional DMARD monotherapy, 72.5%  Combination DMARDs, 3.6%  bDMARD, 1.2% | - Median age (IQR), years: 58 (47–67) - Female, 77.4% - Median disease duration (IQR), days: 107 (77–188) | Biomarkers   - Serum OPG - Serum COMP - Serum CTx-I - Urine CTx-II - Serum YKL-40 - Serum MMP-3 - Serum CRP | MRI images and conventional radiographs scored according to RAMRIS and vdHS; disease activity assessed by DAS28  Progression was defined as increase ≥1 vdHS unit (hands only) | 1 year |
| Tengstrand *et al*., 2004[33]  Sweden  Prospective observational study  Full publication | Early RA patients   - Disease duration <12 months - 1987 ACR criteria - Diagnosed by rheumatologists | 844  (Larsen score baseline subgroup 329) | Mixed population?  DAS28, mean: 5.1 (SD, 1.3)  Larsen score baseline subgroup:  Men, 5.0 (SD, 1.2)  Women, 5.2 (SD, 1.3) | NR | - Mean age (SD), years: 56.6 (15.9) - Female, 76.7% - Disease duration (SD), months: 6.1 (3.0) - CRP, median (IQR), mg/L: 20 (7–44) - DAS28, mean (SD): 5.1 (1.3) - HAQ score, median (IQR): 0.88 (0.50–1.38) | Demographic variables   - Sex - Age - Disease duration - Reported heredity - Presence of the ‘shared epitope’ - Current smoking - RF - CRP - DAS28 - HAQ - Presence of radiographic changes by ACR criteria - Larsen score | Disease activity measured by DAS28  Functional status measured by HAQ  Radiographic outcomes defined by the Larsen scoring system at 2 years of follow-up  Progression not defined | 2 years |
| van Steenbergen *et al*., 2015[34]  Netherlands  Retrospective cohort study  Full publication | Early RA patients   - Disease duration ≥5 years - 1987 ACR criteria for RA - RA confirmed at physical examination and symptom duration ≤2 years | 426 | NR | % naïve/ treated, NR  Conventional DMARD therapy initiated in all patients enrolled after 1996 | - Mean age (SD), years: 56.6 (15.3) - Female, 68.1% - Symptom duration, median (IQR), months: 4.4 (2.4–8.6) - SJC66, median (IQR): 8 (4–14) - ESR, median (IQR), mm/h: 33.0 (18.0–55.0) | Genetic variants   - 17 risk alleles, including *rs4810485* in CD40 and *rs7607479* in SPAG16 | Disease severity defined as radiographic progression in vdHS score over 6 years, assessed as continuous outcome, categorized as no/little, moderate, or severe progression | 6 years |

^a^Moderate RA defined as: described as ‘moderate’ in the publication or DAS28 reported as >3.2–≤5.1.

^b^The authors of this study do provide a breakdown of which conventional DMARD combinations were used in the study; not clear whether each combination relates to a different patient and no total is given.

ACR, American College of Rheumatology; AKA, anti-keratin antibody; AMRICS, Auckland MRI cartilage score; APF, anti-perinuclear factor; ASPIRE, Active-Controlled Study of Patients Receiving Infliximab for the Treatment of Rheumatoid Arthritis of Early Onset; bDMARD, biological disease-modifying anti-rheumatic drug; BeST, Behandel Strategieen; BMI, body mass index; CAMERA, Computer Assisted Management in Early Rheumatoid Arthritis study; CCP, cyclic citrullinated peptide antibody; CD40, cluster of differentiation 40; CDAI, Clinical Disease Activity Index; COMP, cartilage oligomeric matrix protein; CRP, C-reactive protein; CTx-I/II, C-terminal cross-linking telopeptide of type I/II collagen; DAS, Disease Activity Score; DAS28, 28-joint Disease Activity Score; DMARD, disease-modifying anti-rheumatic drug; ESPOIR, Etude et Suivi des POlyarthrites Indifférenciées Récentes; ESR, erythrocyte sedimentation rate; EULAR, European League Against Rheumatism; GSUS, grey-scale ultrasound; HAQ, Health Assessment Questionnaire; HLA, human leukocyte antigen; Ig, immunoglobulin; IQR, Interquartile range; JSN, joint space narrowing; MBDA, multi-biomarker disease activity; M-CDAI, modified Clinical Disease Activity Index; M-DAS28, modified 28-joint Disease Activity Score; M-HAQ, modified Health Assessment Questionnaire; MMP, matrix metalloproteinase; MRI, magnetic resonance imaging; M-SDAI, modified Simple Disease Activity Index; M-vdHS, modified van der Heijde–Sharp; MTX, methotrexate; NR, not reported; OMERACT, Outcome Measures in Rheumatology; OPG, osteoprotegerin; PDUS, power Doppler ultrasound; RA, rheumatoid arthritis; RAMRIS, Rheumatoid Arthritis Magnetic Resonance Imaging Scoring System; RCT, randomized controlled trial; RF, rheumatoid factor; Rf, risk factor; RRP, rapid radiologic progression; SCQM, Swiss Clinical Quality Management; SD, standard deviation; SDAI, Simple Disease Activity Index; SENS, Simple Erosion Narrowing Score; SJC, swollen joint count; SONAR, Swiss Sonography in Arthritis and Rheumatism; SPAG16, sperm-associated antigen 16; SWEFOT, Swedish Farmacotherapy; TJC, tender joint count; TEMPO, Trial of Etanercept and Methotrexate with radiographic Patient Outcomes; TNF, tumour necrosis factor; U/L, upper/lower; vdHS, van der Heijde–Sharp; YKL-40, human cartilage glycoprotein 39.

### **Disease activity progression: study information**

SUPPLEMENTARY TABLE S4 Disease activity progression: study information

| **Reference** | **Patient inclusion criteria** | ***N*** | **Mixed population/ moderate only^a^** | **DMARD history** | **Population characteristics** | **Markers assessed** | **Outcomes assessed/definition of progression** | **Duration of follow-up** |
| --- | --- | --- | --- | --- | --- | --- | --- | --- |
| Bakker *et al*., 2012[9]  USA  Prospective cohort study  Full publication | RA patients   - From CAMERA cohort - 1987 ACR criteria for RA - Age >16 years - Treated with MTX ± ciclosporin | 74 | Mixed population  Baseline DAS28, mean (SD): 5.6 (1.0) | Methotrexate, 100% | - Serum samples from a subgroup in CAMERA cohort - Female: 52 (70%) - Mean (SD) age, years: 53 (15) - RF+: 50 (68%) - TJC28, mean (SD): 9 (6) - SJC28, mean (SD): 10 (5) - ESR, mean (SD), mm/h: 39 (29) | - MBDA test | Clinical disease activity as measured by DAS28-CRP  Radiographic progression, >0 vdHS units over 2 years | 2 years |
| Barra *et al*., 2013[10]  Multicentre prospective observational cohort study  Canada, US and UK | RA patients   - Age >16 years - 6 weeks to 12 months of persistent synovitis - ≥2 swollen joints or 1 swollen MCP/PIP - ≥1 of: RF+, anti-CCP +, morning stiffness >45 min, response to NSAIDs, or a painful MTP squeeze test - Patients were followed up every 3 months using a standard protocol - Treatment with DMARD, NSAID, corticosteroids (oral, intramuscular and/or intraarticular), and biologic agents based on physician’s discretion with the aim of obtaining zero swollen joints - Baseline and 12-month follow-up values for RF and anti-CCP | 321 | Moderate only  Baseline mean (SD) DAS28: 4.83 (1.62) | 309 (9.6) | Anti CCP population (*n* = 342); RF, *n* = 520   - Mean age (SD), years: 52 (13) - Female: 78% - Mean symptom duration (SD), months: 6.5 (3.2) - anti CCP population (*n*= 321): DAS28: 4.83 (1.62) - RF population (*n* = 496): DAS28: 4.78 (1.53) | - RF+ or anti-CCP+ | Disease activity determined using the DAS28; remission defined as DAS28 <2.6. Patient function evaluated using the HAQ | 12–24 months |
| Gonzalez-Alvaro *et al*., 2011[17]  Spain  Prospective longitudinal study  Full publication | RA patients   - To be enrolled in the EAC, patients had ≥2 swollen joints for at least 4 weeks and symptoms for <1 year - Met 1987 ACR criteria for RA (71%) or with chronic undifferentiated arthritis (29%) | 171 | Mixed population  DAS28 baseline median (range), 4.5 (3.3–5.7) | % naïve/ treated, NR | - Female: 76.4% - Median age (range), years: 53 (42–66) - Median disease duration (range), years: 6 (4.2–9.0) - HAQ score at baseline (range): 1 (0.5–1.62) | - IL-15 - RF - Anti-CCP | Disease activity assessed by the DAS28-ESR  Progression not defined | 2 years |
| Kiely *et al*., 2011[20]  UK  Prospective cohort study  Full publication | RA patients   - From Early RA Network (ERAN) - Patients with years 2 and 3 DAS28 and HAQ outcome data by February 2010 | 418 | Mixed population  Baseline DAS28, *n* (%): <3.2, 161 (38.5); 3.2–5.1, *n* = 170; >5.1, *n* = 87 | NR | - Median age, years: 57 - Female: 72% - RF+: 61% - Erosive on plain X-rays of hands and feet: 28% | - DAS28 | DAS28 score and HAQ disability index score  Progression not defined | ≥2 years (subgroup of 302 patients had 3 years of follow-up) |
| Mogosan *et al.,* 2016[26]  Romania  Prospective observational cohort study  Abstract | RA patients   - From Romanian Registry of Rheumatic Diseases - Patients for whom the following measurements were available: EQ-5D, HAQ score, DAS28 and SDAI | 777 | Moderate only  Baseline DAS28, mean (SD): 3.66 (1.52) | % naïve/ treated, NR  Etanercept, 30.6%  Adalimumab, 22.7%  Infliximab: original, 6.3%; biosimilar, 0.7%  Rituximab, 26.3% | - Mean age (SD), years: 58.7 (12.4) - Female: 84.4% - Disease duration (SD), years: 14.1 (8.3) - Retired, %, 77.1% - HAQ, mean (SD): 1.14 (0.64) - EQ-5D, mean (SD): 0.61 (0.31) | Variables including:   - EQ-5D - DAS28 - SDAI - HAQ - disease duration | Disease activity measured by DAS28  Progression not defined | NR |
| Ozmen *et al*., 2014[29]  Turkey  Randomized controlled trial  Abstract | RA patients   - ACR classification criteria - ≥ 6 months of follow-up - Patients with infectious disease, major surgery, severe trauma, malignant disease or serious cardiovascular disease at diagnosis, during assessment or 1 month before assessment were excluded | 43 | Mixed population  Baseline DAS28 <3.2, 14 (33%)  Baseline DAS28 ≥3.2,  29 (67%) | NR | - Female: 31 (72%) - Mean (SD) age, years: 54 (11) - Mean (SD) disease duration, months: 41 (29) - NLR at diagnosis, mean (SD): 2.44 (1.03) - DAS28, mean (SD): 3.64 (0.85) - HAQ score, mean (SD): 0.90 (0.80) | Clinical variables:   - NLR - complete blood count (neutrophil/lymphocyte count) - ESR - CRP | Disability progression-defined disease activity (DAS28), HAQ score and extent of joint deformity (by physical examination) | ≥6 months |
| Predeteanu *et al*., 2009[30]  Romania  Retrospective cohort study  Full publication | RA patients   - 1987 ACR criteria for RA | 111 | Mixed population | NR | - Mean age (SD), years: 54.61 (12.31) - Female: 87% | Clinical biomarkers   - Anti-CCP - CRP - RF | DAS using four variables: number of tender joints; number of swollen joints; ESR; and assessment of disease activity  Progression not defined |  |
| Tengstrand *et al*., 2004[33]  Sweden  Prospective observational study  Full publication | Early RA patients   - Disease duration <12 months - 1987 ACR criteria - Diagnosed by rheumatologist | 844  (Larsen score baseline subgroup = 329) | Mixed population?  DAS28, mean: 5.1 (SD, 1.3)  Larsen score baseline subgroup:  Men: 5.0 (SD, 1.2)  Women: 5.2 (SD, 1.3) | NR | - Mean age (SD), years: 56.6 (15.9) - Female: 76.7% - Disease duration (SD), months: 6.1 (3.0) - CRP, median (IQR), mg/L: 20 (7–44) - DAS28, mean (SD): 5.1 (1.3) - HAQ score, median (IQR): 0.88 (0.50–1.38) | Demographic variables   - Sex - Age - Disease duration - Reported heredity - Presence of the ‘shared epitope’ - Current smoking - RF - CRP - DAS28 - HAQ - Presence of radiographic changes by ACR criteria   Larsen score | Disease activity measured by DAS28  Functional status measured the HAQ  Radiographic outcomes defined by Larsen score at 2 year follow-up  Progression not defined | 2 years |
| Zhao *et al*., 2015[35]  UK  Prospective longitudinal cohort study  Abstract | Early RA patients | 126 | Mixed population  DAS ≤5.1,  *n* = 70 (56%) | % naïve/ treated, NR  Triple DMARD therapy:  19 (15%) | - Stratified into three categorical anxiety depression variables (none, moderate, extreme) - Mean age (SD), years: none, 58 (15.0); moderate, 55.7 (16.9); extreme, 54.4 (16.2) - Female: none, 61.8%; moderate, 83.3%; extreme, 71.4% - Median DAS28 (IQR): none, 4.8 (3.9–5.5); moderate, 4.8 (4.3–5.9); extreme, 6.4 (5.9–7.4) - Median HAQ (IQR): none, 0.75 (0.25–1.25); moderate, 1.25 (0.875–1.5); extreme, 2.0 (1.125–2.125) | Demographic variables examining anxiety and depression derived from detailed questionnaire recording self-rated quality of life, EQ-5D | Progression defined as change in DAS28 and HAQ score | 1 year |

^a^Moderate RA defined as: described as ‘moderate’ in the publication or DAS28 score reported as being >3.2–≤5.1.

ACR, American College of Rheumatology; CAMERA, Computer Assisted Management in Early Rheumatoid Arthritis study; CCP, cyclic citrullinated peptide antibody; CRP, C-reactive protein; DAS, Disease Activity Score; DAS28, 28-joint Disease Activity Score; DMARD, disease-modifying anti-rheumatic drug; EAC, Early Arthritis Clinic; EQ-5D, 5-dimension EuroQol questionnaire; ESR, erythrocyte sedimentation rate; HAQ, Health Assessment Questionnaire; IL, interleukin; IQR, interquartile range; MBDA, multi-biomarker disease activity; MCP, metacarpophalangeal joint; MTP, metatarsophalangeal joint; MTX, methotrexate; NLR, neutrophil:lymphocyte ratio; NR, not reported; NSAID, non-steroidal anti-inflammatory drug; PIP, proximal interphalangeal joint; RA, rheumatoid arthritis; RF, rheumatoid factor; SD, standard deviation; SDAI, Simple Disease Activity Index; SJC, swollen joint count; TJC, tender joint count; vdHS, van der Heijde–Sharp.

### **Other outcomes: study information**

SUPPLEMENTARY TABLE S5 Other outcomes: study information

| **Reference** | **Patient inclusion criteria** | ***N*** | **Mixed population/ moderate only^a^** | **DMARD history** | **Population characteristics** | **Markers assessed** | **Outcomes assessed/ definition of progression** | **Duration of follow-up** |
| --- | --- | --- | --- | --- | --- | --- | --- | --- |
| Bykerk *et al*., 2010[11]  Canada  Prospective observational cohort study  Abstract | Early inflammatory arthritis patients   - Age >16 years - 6–52 weeks of persistent synovitis - ≥2 effused joints or 1 swollen MCP/PIP - ≥1 of: RF+, anti-CCP+, morning stiffness >45 minutes, response to NSAIDs or a painful MTP squeeze test - Patients were treated with ≥2 DMARDs before being eligible for biologic therapy | 1146 | Moderate only (DAS28 ESR – 4.9 + 1.6) | MTX, 50% Combination DMARDs, 35%  Biologics, 8% | - Mean age (SD), years: 52 (16) - Female: 73% - Symptom duration, months: 5.5 - DAS28 ESR, mean (SD), mm/h: 4.9 (1.6) - Erosions at baseline: 25% - Treatment within 1 year: 27% oral glucocorticoids, 50% MTX, 35% combination DMARDs, 8% biologics - Met 1987 criteria for RA: 73% | 13 clinical variables including:   - age - sex - DAS28 at baseline and 3 months - MTX use at doses ≥20 mg by 3 months - baseline erosions - socioeconomic status - active smoking status - anti-CCP+ - RF+ - higher than normal CRP/ESR - SJC (0–28) - TJC (0–28) - symptom duration | Progression defined as use of biologics within 1 year | Median 0.75 years (up to 8 years) |
| Michaud *et al*., 2011[25]  USA  Prospective longitudinal cohort study  Full publication | RA patients   - Diagnosis by patient’s rheumatologist - Patients recruited to RA database to start a biologic at enrolment were excluded (severity bias) - Moderate to severe RA identified comparing patient-reported severity with PAS score, a composite of HAQ, VAS pain and VAS patient global assessment scores | 18,485 | Mixed population  Moderate or severe, 52.2% | % treated/ naïve, NR | - Mean age, years: ~ 60 - Female: 76.7% - Disease duration, years: ~ 12 - Patient global assessment, mean (SD): 3.6 (2.5) - DAS28 CRP, median (IQR): 3.3 (2.3–4.3) - HAQ score at entry (SD): 1.06 (0.73) - Mean (SD) number of DMARDs/biologics used at entry: 2.7 (1.9) | Nine demographic variables   - Age - Sex - College education - Smoking status - BMI - Comorbidity type - Number of comorbidities - Year of RA onset - Moderate or severe RA | Disability progression defined as annual rate of HAQ FDI progression | Mean (SD), 3.7 (3.2) years  Range, 0.5–11 years |
| Nikiphorou *et al.,* 2015[28]  UK  Observational cohort study  Abstract | RA patients   - Patients from the Early RA Study (ERAS) and the Early RA Network (ERAN) | Low moderate, 522  High moderate, 426 | Moderate only:   - low-moderate (mean DAS28 during years 1–5: >3.2–4.19): baseline mean (SD): 4.8 (1.2) - high-moderate (mean DAS28 during years 1–5: 4.1–5.1): baseline mean (SD): 5.3 (1.2) | NR | Low moderate   - Age at onset (SD), years: 56 (14) - Female: 69% - Hb, mean (SD): 12.9 (12.8) g/dL - HAQ, mean (SD): 1.07 (0.71)   BMI, mean (SD): 26.5 (5.3) kg/m^2^  High-moderate   - Age at onset (SD), years: 56 (14) - Female: 77% - Hb, mean (SD): 12.6 g/dL - HAQ, mean (SD): 1.29 (0.71) - BMI, mean (SD): 26.3 (5.2) kg/m^2^ | - DAS28 | Intermediate and major joint surgery, used as a surrogate marker of joint failure | 5 years |

^a^ Moderate RA defined as: described as ‘moderate’ in the publication or DAS28 score reported as being >3.2–≤5.1.

BMI, body mass index; CCP, cyclic citrullinated peptide; CRP, C-reactive protein; DAS28, 28-joint Disease Activity Score; DMARD, disease-modifying anti-rheumatic drug; ESR, erythrocyte sedimentation rate; FDI, Functional Disability Index; HAQ, Health Assessment Questionnaire; Hb, haemoglobin; HRQOL, health-related quality of life; MCP, metacarpophalangeal joint; MCS, Mental Component Score (Short-Form 36); MTP, metatarsophalangeal joint; MTX, methotrexate; NR, not reported; NSAID, non-steroidal anti-inflammatory drug; PAS, Patient Activity Scale; PCS, Physical Component Score (Short-Form 36); PIP, proximal interphalangeal joint; RA, rheumatoid arthritis; RF, rheumatoid factor; SD, standard deviation; SF-36, Short-form 36 Health Survey; SJC, swollen joint count; TJC, tender joint count; VAS, visual analogue scale.

###

### **Deprioritized studies**

SUPPLEMENTARY TABLE S6 Summary of deprioritized studies (mixed RA population)

| **Factor** | **Reference(s)** | **Results** |
| --- | --- | --- |
| **Radiographic progression** | | |
| Larsen score | Tengstrand et al., 2004[33] | Low Larsen score at baseline was predictive of a worse Larsen score at 2 years of follow-up |
| Antibody status | Hayem *et al*., 1999[19] | Presence of anti-Sa antibodies at baseline was a significant risk factor for the development of severe destructive RA (Larsen score ≥4) after 6 years (OR, 5.41; 95% CI, 1.76–16.6) |
|  | del Val del Amo *et al*., 2006[16] | After 6 years of follow-up, patients with anti-CCP antibodies had developed significantly more severe radiological damage (measured by change in vdHS score) than those without. Presence of anti-CCP antibodies was predictive of disease progression as measured by changes in vdHS score |
| Genetic biomarkers | van Steenbergen *et al*., 2015[34] | HLA-DRB1, CD40, IL-15, DKK-1, IL2RA, GRZB IL-4R, SPAG16, C5orf30, MMP-9, rs1465788 and OPG together were able to explain 12–18% of disease progression (increase of 1–5 points in vdHS score). None was an accurate predictor of disease progression alone  Note: population not explicitly defined, so assumed to be mixed in order to be conservative. |
|  | Syversen *et al*., 2009[32] | Serum CTx-I (*P* = 0.049) and urine CTx-II (*P* = 0.043) predictive of structural damage progression (as measured by change in vdHS) |
|  | Hambardzumyan *et al*., 2015[18] | 20.9% of patients with a high MBDA had RRP. Progression occurred in 14%, 15%, 14% and 15% of patients with low CRP, moderate CRP, moderate DAS28-CRP and moderate DAS28-ESR at baseline, respectively. MBDA score as a continuous variable was a strong independent predictor of RRP after 1 year |
|  | Markusse *et al*., 2014[23] | Correlation between MBDA score and subsequent disease progression as measured by increase in vdHS score over 1 year. Higher MBDA scores measured at baseline were associated with an increased risk for vdHS progression in the subsequent year, adjusted for ACPA positivity and DAS (RR 1.039, 95% CI 1.018–1.059; Table). For each 10-unit increase in baseline MBDA score, there was a 1.47-fold increase in the risk of progression at Year 1. |
|  | de Punder *et al*., 2015[15] | More risk factors at baseline (anti-CCP antibody presence, high ESR, degree of erosion) in addition to a higher DAS28 resulted in a higher probability of joint damage progression (>5 Ratingen point increase) after 3 years of follow-up |
|  | De Cock *et al*., 2014[13] | ASPIRE CRP, ASPIRE ESR, BeST, SWEFOT1, SWEFOT2 and ESPOIR matrices assessed for prediction of disease progression by increases in vdHS score. Only ASPIRE CRP had moderate predicting value at year 1, 2 and >2 |
| Modified DAS scores | Couderc *et al*., 2015[12] | Ability of the various modified scores to predict radiographic progression after 1 year was the same as baseline:   - M-DAS28-ESR and DAS28-ESR (AUC, 0.53 [0.49–0.58] vs 0.53 [0.48–0.58], respectively) - M-DAS28-CRP and DAS28-CRP (AUC, 0.54 [0.49–0.59] vs 0.52 [0.47–0.57], respectively) - M-SDAI and SDAI (AUC 0.52 [0.47–0.57] vs 0.51 [0.46–0.56], respectively) - M-CDAI and CDAI (AUC, 0.51 [0.47–0.56] vs 0.50 [0.45–0.55], respectively) |
| **Disease progression** | | |
| Antibody status | Gonzalez-Alvaro *et al*., 2011[17] | Anti-CCP antibody levels significantly associated with DAS28 during 2 years of follow-up |
|  | del Val del Amo *et al*., 2006[16] | Presence of anti-CCP antibodies predictive of disease progression, as measured by changes in HAQ and vdHS score |
|  | Predeteanu *et al*., 2009[30] | Anti-CCP antibodies correlated with DAS at 6 months of follow-up |
| Genetic markers | Bakker *et al*., 2012[9] | Significant correlation between MBDA score and progression measured by DAS28-CRP score (*r* = 0.72; *P* < 0.001) |
|  | Gonzalez-Alvaro *et al*., 2011[17] | High serum IL-15 at baseline was significantly associated with a high DAS28 during 2 years of follow-up |
| Anxiety and depression | Zhao *et al*., 2015[35] | Moderate or extreme baseline anxiety and depression slightly predictive of a reduced likelihood of DAS28 remission (OR, 0.38; 95% CI, 0.18–0.81) and good EULAR criteria response (OR, 0.41; 95% CI, 0.13–1.03) at 12 months |
| Neutrophil: lymphocyte ratio (NLR) | Ozmen *et al*., 2014[29] | There was no relationship between mean NLR at diagnosis and mean DAS28 and HAQ score  There was a weak correlation between mean NLR and mean CRP level at diagnosis |
| **Other progression indicators** | | |
| Change in functional disability | Michaud *et al*., 2011[25] | Age is an important predictor of the rate of loss of functional status (measured by HAQ FDI) |

ACPA, anti-citrullinated protein antibodies, ASPIRE, Active-Controlled Study of Patients Receiving Infliximab for the Treatment of Rheumatoid Arthritis of Early Onset; AUC, area under curve; BeST, Behandel Strategieen; CCP, cyclic citrullinated peptide; CI, confidence interval; CRP, C-reactive protein; DAS, Disease Activity Score; DAS28, 28-joint Disease Activity Score; DAS28-CRP 28-joint Disease Activity Score using C-reactive protein; CTx-I/II, C-terminal cross-linking telopeptide of type I/II collagen; ESPOIR, Etude et Suivi des POlyarthrites Indifférenciées Récentes; ESR, erythrocyte sedimentation rate; FDI, Functional Disability Index; HAQ, Health Assessment Questionnaire; HLA, human leukocyte antigen; IL, interleukin; MBDA, multi-biomarker disease activity; MMP, matrix metalloproteinase; NR, not reported; OPG, osteoprotegerin; OR, odds ratio; RA, rheumatoid arthritis; RF, rheumatoid factor; RR, risk ratio; SDAI, Simplified Disease Activity Index; SF-36, Short-form 36 health survey; SWEFOT, Swedish Farmacotherapy; vdHS, van der Heijde–Sharp.

### **Disease activity progression: Results**

SUPPLEMENTARY TABLE S7 Radiographic progression: results

| **Reference** | **Results** | **Authors’ conclusions** |  |
| --- | --- | --- | --- |
| Alemao *et al*., 2014[8] | - Baseline factors associated with RRP in logistic regression - RF+ or anti-CCP+ antibody (OR, 3.35; 95% CI, 1.41–7.99) - Duration of RA <2 years (OR, 0.22; 95% CI, 0.07–0.64) - Under to normal weight (OR, 4.88; 95% CI, 1.82–13.11) - DAS28-CRP (OR, 1.24; 95% CI, 1.02–1.52) - vdHS score (OR, 1.01; 95% CI, 1.00–1.01) | RRP in RA can be predicted based on baseline seropositivity, body weight, disease duration, DAS28-CRP and vdHS score. Further validation of the model with other data sets is required to confirm the findings |  |
| Alemao *et al.,* 2016[7] | - Presence of anti-CCP but not RF was predictor for erosive disease (OR, 2.72 [95% CI, 1.77–4.18] and 1.36 [0.88–2.08] respectively) and for low disease activity (SDAI <3.3) (0.37 [0.21–0.66] and 1.45 [9% CI, 0.82–2.56] respectively) - Anti-CCP+ patients with erosions had lower odds of SDAI remission compared with all other patients (0.19 [0.10–0.37]) | Anti-CCP has a stronger association with erosions and disease activity. The presence of anti-CCP-antibody and erosions (vs absence) is associated with higher disease activity and lower odds of remission |  |
| Bakker *et al*., 2012[9] | - At baseline neither the MBDA score (OR, 1.018; 95% CI, 0.988–1.049; *P* = 0.25) nor DAS28-CRP (OR, 1.041; 95% CI, 0.645–1.680; *P* = 0.87) was a predictor of radiographic progression - After inclusion of other known predictors for poor long-term outcome (RF and baseline damage) in a multivariate logistic regression model, the baseline MBDA score was borderline significant as an independent predictor for progression of joint damage (OR, 1.033; 95% CI, 0.995–1.072, *P* = 0.09) next to RF (OR, 1.768; 95% CI, 0.396–7.885, *P*= 0.46) and baseline joint damage (OR, 4.213; 95% CI, 0.878–20.211; *P* = 0.07) - Neither MBDA score nor clinical variables were predictive of radiographic progression | This multi-biomarker test performed well in the assessment of disease activity in patients with RA in the CAMERA study. With further validation, this test could be used to complement currently available disease activity measures and to improve patient care and outcomes |  |
| Couderc *et al*., 2015[12] | - Ability to predict radiographic progression after 1 year was the same as baseline - M-DAS28-ESR and DAS28-ESR (AUC, 0.53 [0.49–0.58] vs 0.53 [0.48–0.58], respectively) - M-DAS28-CRP and DAS28-CRP (AUC, 0.54 [0.49–0.59] vs 0.52 [0.47–0.57], respectively) - M-SDAI and SDAI (AUC 0.52 [0.47–0.57] vs 0.51 [0.46–0.56], respectively) - M-CDAI and CDAI (AUC, 0.51 [0.47–0.56] vs 0.50 [0.45–0.55], respectively) | M-DAS (M-DAS28-ESR, M-DAS28-CRP, M-CDAI and M-SDAI), based on clinical measures of disease activity, did not prove superiority to original disease activity scores in ability to predict structural progression in patients with RA |  |
| De Cock *et al*., 2014[13] | - Four patients developed RRP in year 1, five in year 2 and four over 2 years - One patient who developed RRP over 2 years was always found in the lowest RRP categories of all matrices - The ASPIRE CRP matrix yielded a moderate predictive value for the three time points while other matrices showed moderate to no predictive value | The predictive performance of the six matrices (ASPIRE CRP/ESR matrices, the BeST matrix, two SWEFOT 1/2 matrices, and the ESPOIR matrix) to detect risk of RRP was modest at best |  |
| De Miguel *et al*., 2015[14] | - Disease activity at any study time by any composite index (DAS28-ESR, DAS28-CRP, SDAI, CDAI or ACR/EULAR criteria) was not significantly associated with radiographic progression - PDUS score ≥1 at baseline and persistence at 6 months were associated with radiographic progression | A 12-joint PDUS score showed better predictive validity for structural damage progression in RA than composite indices of disease activity |  |
| de Punder *et al*., 2015[15] | - More risk factors at baseline, as well as a higher DAS, resulted in a higher probability of joint damage progression in a dose-dependent way - The probability of joint damage progression increased with increasing DAS and increasing number of risk factors | In patients without the risk factors, a moderate DAS as a treatment target is stringent enough to prevent joint damage progression; in patients with all three risk factors (presence of anti-CCP antibody, acute-phase response and erosions at baseline), a low DAS treatment target is not stringent enough to limit the risk of joint damage |  |
| del Val del Amo *et al*., 2006[16] | - There was a significant correlation between anti-CCP levels and CRP - The mean value of DAS28 in anti-CCP+ patients was 4.31 (SD, 1.27) compared with 3.30 (SD, 1.55) in anti-CCP− patients - SENS was significantly greater in anti-CCP+ than in anti-CCP− patients - There was no correlation between M-HAQ score and anti-CCP levels | The prevalence of anti-CCP antibody was higher among patients with higher vs lower disease activity.  Patients with higher levels of anti-CCP had more aggressive disease, with greater activity (elevated DAS28 and CRP) and more severe radiological damage (more erosions and higher radiological damage, SENS) than those with lower anti-CCP levels |  |
| Fautrel *et al*., 2015[3] | - The matrix risk model showed that RF positivity and CRP >40 mg/L at baseline were significantly associated with SRP after 2 and 3 years - Baseline erosion score was not predictive of SRP | Patients with moderate RA receiving MTX treatment may exhibit radiographic progression, particularly those with both high CRP and RF+ at baseline, after 2 and 3 years of MTX treatment |  |
| Hambardzumyan *et al*., 2015[18] | - 20.9% of patients with a high MBDA had RRP - Progression occurred in 14%, 15%, 14% and 15% of patients with low CRP, moderate CRP, moderate DAS28-CRP and moderate DAS28-ESR at baseline, respectively - MBDA score as a continuous variable was a strong independent predictor of RRP after 1 year | In DMARD-naïve patients with early RA, a low or moderate MBDA score at baseline was associated with a very low risk of RRP after 1 year |  |
| Hayem *et al*., 1999[19] | - In patients with long-standing RA, significant associations were found between the presence of anti-Sa and RF (*P* < 0.0001), AKA (*P* < 0.0001), APF (*P* < 0.0001) and HLA DRB1*04 or 1 (*P* < 0.01) - Those with destructive disease were more likely to test positive for anti-Sa antibody (66.6%) than were those without destructive disease - The anti-Sa-antibody test was sensitive (68.4%), and was also the test with the highest specificity (79%) compared with other serological biomarkers for RA | Immunoblot-detected IgG anti-Sa is a sensitive serological biomarker for RA in patients with severe radiographic damage |  |
| Kroot *et al*., 2000[21] | - Patients with anti-CCP had developed significantly more severe radiological damage after 6 years of follow-up - In multiple regression analysis, radiological damage after 6 years of follow-up was predicted by IgM-RF status, radiological score at entry and anti-CCP status - Functional disability (HAQ) was predicted by sex, age at entry, IgM-RF status and DAS | In almost 70% of RA patients, anti-CCP is present at the early stages of disease  Anti-CCP+ patients developed significantly more severe radiological damage than patients who were anti-CCP− |  |
| Li *et al*., 2016[22] | - The frequency and severity of radiographic progression increased as MBDA scores became higher within the high range (17.4 for change in vdHS score >5 with MBDA score ≥60) - In multivariate analyses, MBDA score had the most significant association with radiographic progression (*P* = 0.002 for change in vdHS score >3; *P* = 0.005 for change in vdHS score >5) - When conventional risk factors such as SJC, CRP and DAS28-CRP were low, MBDA score significantly differentiated risk for progression | MBDA score enhanced the ability of conventional risk factors (i.e. serological status, SJC [0–28], CRP and DAS28-CRP) to predict radiographic progression in patients with established RA receiving non-biologic DMARDs |  |
| Markusse *et al*., 2014[23] | - MBDA scores distinguished between patients who developed radiographic progression (increase in vdHS score ≥5 points) and patients who did not - Categorized high MBDA scores were also correlated with radiographic progression - For patients with recent-onset RA who received treat-to-target therapy, MBDA scores at baseline and 1-year predicted radiographic damage progression in the subsequent year | MBDA scores predict radiographic progression at baseline and during disease course |  |
| McQueen *et al*., 2014[24] | - AMRICS scores correlated with the OMERACT JSN and X-ray JSN - MRI cartilage, synovitis and osteitis scores predicted 3-year AMRICS scores - Baseline radial osteitis predicted increased cartilage scores at the radiolunate and radioscaphoid joints - Synovitis at radioulnar, radiocarpal and intercarpal–carpometacarpal joints also influenced 3-year cartilage scores | MRI cartilage damage progression is preceded by osteitis and synovitis but is most influenced by pre-existing cartilage damage |  |
| Moller *et al*., 2016[27] | - A significantly higher proportion of patients (*P* < 0.05) with GSUS or PDUS scores higher than GSUS and PDUS lower thresholds (20%, 30% or 50%) had radiographic damage progression | Ultrasound-detected synovitis may predict radiographic damage progression in RA |  |
| Sundlisater *et al*., 2016[31] | - In univariate models, *P* < 0.25 for sex, age, smoking status, RF, tender joints, 44 SJC, ESR, total GSUS score, total PDUS score and vdHS at baseline; *P* > 0.25 for BMI, disease duration <3 months, anti-CCP and patient’s global assessment - In the multivariate model, RF positivity (OR, 2.27, *P* = 0.022), total vdHS (OR, 1.08, *P* = 0.017) and GSUS score (OR, 1.03 per point, *P* = 0.019) were independent baseline predictors of radiographic progression at 24 months | RF positivity, radiographic joint damage and GSUS score were independent baseline predictors of joint damage in patients with early RA receiving aggressive treatment aiming for remission. This indicates that further individualization of treatment based on risk factors might be needed to optimize disease outcomes and in treat-to-target strategies | |
| Syversen *et al*., 2009[32] | - Baseline serum CTx-I predicted progression in joint destruction assessed by MRI and conventional radiographs, whereas urine CTx-II was a predictor of progression in vdHS score but not RAMRIS - Serum YKL-40 and MMP-3 in addition to CRP were consistently associated with both MRI inflammation (synovitis and bone marrow oedema) and DAS28 at baseline and in longitudinal analyses | Levels of serum CTx-I and urine CTx-II were predictors of progressive joint destruction, whereas serum MMP-3 and YKL-40 were merely biomarkers of joint inflammation |  |
| Tengstrand *et al*., 2004[33] | - At 2-years of follow-up, women still had higher DAS28 and HAQ scores than men - Larsen score showed no sex difference at study entry or after 2 years - Higher DAS28 and HAQ scores at entry were more strongly correlated with severe disease at follow-up in women than in men | Prognostic biomarkers differed between the sexes in the ability to predict outcome at 2 years. RF positivity was more important in men and disease activity was more important in women |  |
| van Steenbergen *et al*., 2015[34] | - The genetic factors together explained 12–18% of the variance in radiographic progression; when added to a model including traditional factors and treatment effects, the genetic factors additionally explained 3–7% of the variance in radiographic progression - The proportion of patients who were correctly classified in the category of radiographic progression increased from 56% to 62%; the net proportion of correct reclassifications was 6% | All genetic severity factors together explained 12–18% of the variance in radiographic progression  Additional use of genetic factors increased the correct classification of patients in severity risk groups |  |

ACR, American College of Rheumatology; AKA, anti-keratin antibody; AMRICS, Auckland MRI cartilage score; APF, anti-perinuclear factor; AUC, area under curve; BeST, Behandel Strategieen; BMI, body mass index; CAMERA, Computer Assisted Management in Early Rheumatoid Arthritis study; CCP, cyclic citrullinated peptide antibody; CDAI, Clinical Disease Activity Index; CI, confidence interval; CRP, C-reactive protein; CTx-I/II C-terminal cross-linking telopeptide of type I/II collagen; DAS, Disease Activity Score; DAS28, 28-joint Disease Activity Score; DMARD, disease-modifying anti-rheumatic drug; ESPOIR, Etude et Suivi des POlyarthrites Indifférenciées Récentes; ESR, erythrocyte sedimentation rate; EULAR, European League Against Rheumatism; GSUS, grey-scale ultrasound; HAQ, Health Assessment Questionnaire; HLA, human leukocyte antigen; Ig, immunoglobulin; JSN, joint space narrowing; MBDA, multi-biomarker disease activity; M-CDAI, modified Clinical Disease Activity Index; M-DAS, modified Disease Activity Score; M-HAQ, modified Health Assessment Questionnaire; M-SDAI, modified Simple Disease Activity Index; MMP, matrix metalloproteinase; MRI, magnetic resonance imaging; MTX, methotrexate; OMERACT, Outcome Measures in Rheumatology; OR, odds ratio; PDUS, power Doppler ultrasound; RA, rheumatoid arthritis; RAMRIS, Rheumatoid Arthritis Magnetic Resonance Imaging Scoring System; RF, rheumatoid factor; RRP, rapid radiographic progression; SDAI, Simple Disease Activity Index; SENS, Simple Erosion Narrowing Score; SJC, swollen joint count; SRP, significant radiographic progression; SWEFOT, Swedish Farmacotherapy; vdHS, van der Heijde–Sharp; YKL-40, human cartilage glycoprotein 39

**Other outcomes: Results**

SUPPLEMENTARY TABLE S8 Disease activity progression: results

| **Reference** | **Results** | **Authors’ conclusions** |
| --- | --- | --- |
| Bakker *et al*., 2012[9] | - The MBDA score had a significant correlation with DAS28-CRP (*P <* 0.001) and an area under the receiver operating characteristic curve for distinguishing remission/low from moderate/high disease activity of 0.86 (*P* < 0.001) using a DAS28-CRP cut-off of 2.7 - After inclusion of other known predictors for poor long-term outcome (RF and baseline damage) in a multivariate logistic regression model, the baseline MBDA score was borderline significant as an independent predictor for progression of joint damage (OR, 1.033; 95% CI, 0.995–1.072; *P* = 0.09) next to RF (OR, 1.768; 95% CI, 0.396–7.885; *P* = 0.46) and baseline joint damage (OR, 4.213; 95% CI, 0.878–20.211: *P* = 0.07) | This multi-biomarker test performed well in the assessment of disease activity in patients with RA in the CAMERA study. With further validation, this test could be used to complement currently available disease activity measures and to improve patient care and outcomes |
| Barra *et al*., 2013[10] | - At 24 months, 15/181 (8%) anti-CCP+ patients became CCP−. A larger number of patients changed from anti-CCP− to anti-CCP+: 13/123 (11%) - For RF, fluctuations were more common: 67/240 (28%) reverted from positive to negative and 21/136 (18%) converted from negative to positive - RF and anti-CCP antibody fluctuations did not predict disease outcomes | RF and anti-CCP antibody have the potential to revert and convert during the early course of RA. Fluctuations in RF and anti-CCP antibody were not associated with clinical outcomes |
| Gonzalez-Alvaro *et al*., 2011[17] | - High serum IL-15 or anti CCP-antibody levels were significantly and independently associated with a higher DAS28 during follow-up - Patients with elevated serum IL-15 had a significantly higher likelihood of receiving intensive treatment | Patients with early RA displaying high baseline serum IL-15 experienced more severe disease and received more intensive treatment |
| Kiely *et al*., 2011[20] | - DAS28 score after 1 year of DMARD therapy was associated with a low DAS28 score (<3.2) and low HAQ score (<1.5) after 2 and 3 years of therapy - Comparing patients with DAS28 scores of <3.2 vs 3.2–5.1 at 1 year, the odds of a low DAS28 score at 2 and 3 years were 7.64 (95% CI, 4.6–12.6) and 4.49 (95% CI, 2.5–7.9) respectively, and the odds of a low HAQ score at 2 and 3 years was 3.47 (95% CI, 2.1–5.6) and 4.92 (95% CI, 2.6–9.0) respectively - Comparing patients with DAS28 scores of 3.2–5.1 vs >5.1 at 1 year, the odds of a low DAS28 score at 2 and 3 years were 2.56 (95% CI, 1.2–5.2) and 3.09 (95% CI, 1.3–7.2), respectively, and the odds of a low HAQ score at 2 and 3 years were 3.19 (95% CI, 1.7–5.7) and 2.5 (95% CI, 1.2–5.1), respectively - Comparing patients with DAS28 scores of 3.2–4.1 vs 4.2–5. 1 at 1 year, the odds of a low DAS28 score at 2 and 3 years were 3.12 (95% CI, 1.5–6.6) and 4.06 (95% CI, 1.7–9.7), respectively, and the odds of a low HAQ score at 2 and 3 years were 2.51 (95% CI, 1.3–4.7) and 2.29 (95% CI, 1.05–4.9), respectively | Ongoing DMARD therapy from year 1 to year 2 or 3 results in a very low likelihood of achieving target DAS28 <2.6 or <3.2 or a target HAQ score <1.5 in patients who have not already achieved this by year 1 |
| Mogosan *et al.,* 2016[26] | - There was a strong negative association between DAS28 (and SDAI) and EQ-5D score (*P* < 0.001). HAQ score positively correlated with DAS28, same as with SDAI (*P <* 0.001) whereas there was a negative association between HAQ and EQ-5D scores (*P* < 0.001) - Disease activity dynamics (change in DAS28 for the last 6 months) had a mild association with HAQ (*r* = 0.1; *P* < 0.05) and a negative association with EQ-5D score (*P* < 0.01) - Patient global assessment of disease activity had a significant association with both HAQ (*P <* 0.001) and EQ-5D scores (*P* < 0.01) - There are significant predictive relationships between DAS28, HAQ and EQ-5D scores: linear regression modelling showed that DAS28 is strongly predicted by EQ-5D (*P* < 0.0001), over 50% of the variability of DAS28 being determined by the variability of EQ-5D. HAQ score has a predictive value for DAS28 (*P* < 0.0001), roughly 30% of the variability of DAS28 being determined by the variability of HAQ - Disease duration did not influence EQ-5D, HAQ or DAS28 scores | Disease activity influences patient wellbeing and functional status. Regression modelling showed that DAS28 could be predicted by the evaluation of EQ-5D and HAQ scores, regardless of the disease duration |
| Ozmen *et al*., 2014[29] | - There was no relationship between mean NLR at diagnosis and mean DAS28 and HAQ score - There was a weak correlation between mean NLR and mean CRP level at diagnosis | There was no association between NLR and disease activity, but there was an association between NLR and CRP at diagnosis |
| Predeteanu *et al*., 2009[30] | - There was a relevant correlation between anti-CCP antibody level and DAS (*r* = 0.437) - Analysis of patients with RA according to the presence or absence of RF and level of anti-CCP antibodies showed that patients with RF had the highest level of anti-CCP antibodies - Statistical analysis of the correlation between anti-CCP antibodies and RF in the group of patients with RA showed a highly relevant correlation (*r* = 0.38) | Anti-CCP antibodies are biomarkers of RA activity, based on their significant correlation with DAS, fibrinogen and CRP |
| Tengstrand *et al*., 2004[33] | - At 2 years of follow-up, women had higher DAS28 and HAQ scores than men - Larsen score showed no sex difference at study entry or after 2 years - Higher DAS28 and HAQ scores at entry were more strongly correlated with severe disease at follow-up in women than in men | Prognostic biomarkers differed between the sexes in ability to predict outcome at 2 years, RF positivity being more important in men and disease activity being more important in women |
| Zhao *et al*., 2015[35] | - Age- and sex-adjusted logistic regression revealed that baseline anxiety and depression predicted a reduced likelihood of DAS28 and good EULAR response at 12 months - Baseline anxiety and depression was not significantly associated with mean change in DAS28 or minimal clinically significant improvement in HAQ score | Anxiety and depression at diagnosis of early RA is a strong predictor of poor outcome (disease activity and disability) at 12 months |

CAMERA, Computer Assisted Management in Early Rheumatoid Arthritis study; CCP, cyclic citrullinated peptide; CI, confidence interval; CRP, C-reactive protein; DAS28, 28-joint Disease Activity Score; DMARD, disease-modifying anti-rheumatic drug; EQ-5D, 5-dimension EuroQol questionnaire; EULAR, European League Against Rheumatism; HAQ, Health Assessment Questionnaire; IL, interleukin; MBDA, multi-biomarker disease activity; NLR, neutrophil:lymphocyte ratio; OR, odds ratio; RA, rheumatoid arthritis; RF, rheumatoid factor; SDAI, Simple Disease Activity Index.

**Other outcomes**

SUPPLEMENTARY TABLE S9 Other outcomes: results

| **Reference** | **Results** | **Authors’ conclusions** |
| --- | --- | --- |
| Bykerk *et al*., 2010[11] | - Of the variables analysed, the only independent factor predicting the use of biologics at 1 year was the DAS28 at baseline (OR, 1.48, *P* < 0.000001) | Of all the classic baseline factors used to determine prognosis in RA in previous studies, baseline DAS28 was the only independent factor predicting use of biologics at 1 year |
| Michaud *et al*., 2011[25] | - Annual HAQ score progression was associated with age, comorbidity, initial severity and treatment - The following variables predicted HAQ score progression: - age >65 years: APR, 0.031 (95% CI, 0.028– 0.034) - heart disease (positive): APR, 0.024 (95% CI, 0.017–0.032) - hypertension (positive): APR, 0.021 (95% CI, 0.018–0.024) - moderate or severe RA (positive): APR, 0.003 (95% CI, 0.001–0.006) - The rate of HAQ score progression for all patients taking biologics was 0.008 (95% CI, 0.005–0.011); the rate of progression for patients with moderate to severe RA taking biologics was 0.001 (95% CI, –0.005 to 0.003) | Age and comorbidities are important predictors of the rate of loss of functional status, and have a stronger effect on HAQ score progression than does biologic treatment |
| Nikiphorou *et al.,* 2015[28] | - In multivariate Cox regression models controlling for age at disease onset, sex, recruitment year, symptom duration, baseline RF, BMI, HAQ, erosions and haemoglobin, high-moderate DAS (HR, 1.80; 95% CI, 1.05–3.11; *P* = 0.034) and high DAS (HR, 2.59; 95% CI, 1.49, 4.52; *P* = 0.001) predicted higher risk for intermediate surgery, unlike low-moderate or low DAS categories - In the case of major joint surgery, low-moderate DAS (HR, 2.07; 95% CI, 1.28–3.33), high-moderate DAS (HR, 2.16; 95% CI, 1.32–3.52) and high DAS (HR, 2.48; 95% CI, 1.50–4.11) all predicted an increasing risk (*P* < 0.005) | Patients who remain in low- or high-moderate DAS in the first 5 years of disease, despite conventional DMARD therapy, have similar risks for joint failure and surgery as those with persistently high DAS. This is highly relevant in health systems where the use of biologic DMARDs is restricted based on DAS thresholds and moderate RA is excluded |

APR, annual progression rate; BMI, body mass index, CI, confidence interval; DAS28, 28-joint Disease Activity Score; DMARD, disease-modifying anti-rheumatic drug; HAQ, Health Assessment Questionnaire; HR, hazard ratio; MCS, Mental Component Score (Short-Form 36); OR, odds ratio; PCS, Physical Component Score (Short-Form 36); RA, rheumatoid arthritis; RF, rheumatoid factor; SF-36, Short Form 36 Health Survey.
